# Supplementary material for: Impairment of the ER/mitochondria compartment in human cardiomyocytes with PLN p.Arg14del mutation
Source: EMBO Mol Med. 2021 May 16;13(6):e13074. doi: 10.15252/emmm.202013074 (PMC8185541; doi:10.15252/emmm.202013074)
Supplement: Supplementary file 1 — Appendix [file EMMM-13-e13074-s011.pdf]

Appendix Figure S1-9

Appendix Figure S1

A 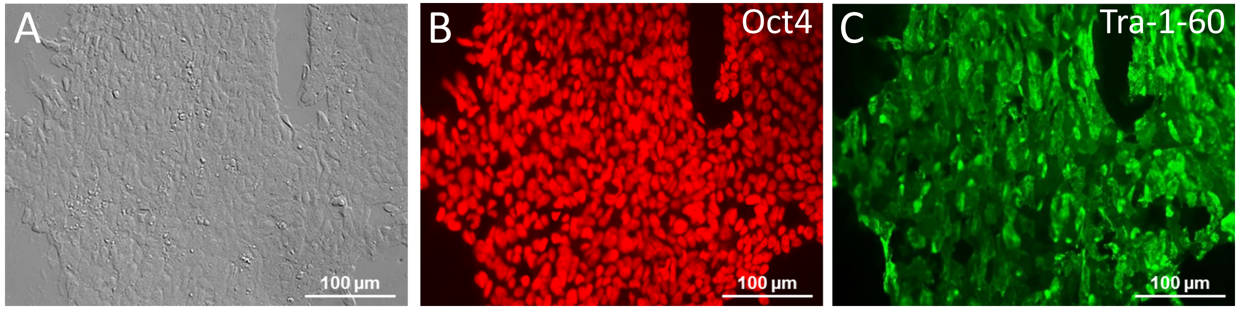

D

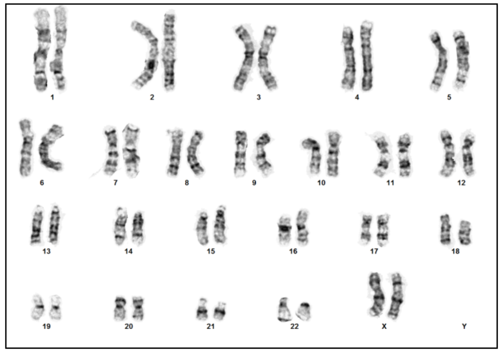

E

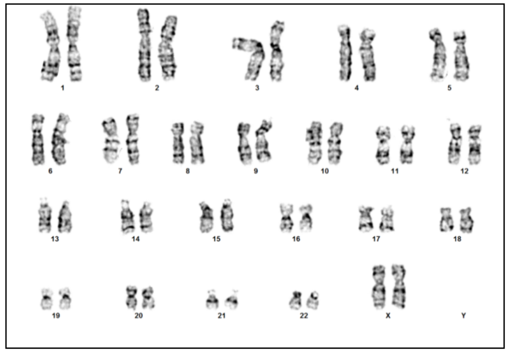

F

Name	Sequence forward (5'-3')	Sequence reverse (5'-3')	PCR product size
OT1	AGCAGGCAGCCCTATTTTCAT	AGAGAGGAGCAAGACAGACTCA	555 bp
OT2	GACATGGCTGATTATATTCTTGCTG	GATCCAGGCTGGCTAAGGTAG	508 bp
OT3	GGGAGCCCACTGATGTGAAG	ACAATCTCAACGTGGAATAGGGA	530 bp
OT4	GGGTGGGCTGAGCCAATAAT	TGAGTATACATTGCTTTGGAGTACA	657 bp
OT5	AGGCACTCGCAAGCTTCTTT	CAGCAGAAGTGTACTACAAAGACC	530 bp
OT6	CCTCATGTATCTGCAGGTGTGT	TGCCACAATGGCTAGTGTATGT	720 bp
OT7	TGTCTCACTCAACATACGTGGT	CTGAGGAAGCAGGAGAGGAGTA	815 bp
OT8	TTGTTCTGCCAGGACCCTAAG	TGAGCACCACAAAATGGGACT	501 bp
OT9	AGGGTGGGTGACTGAGTGTT	ACTTGTCATGGGAGTCGCTTT	587 bp
OT10	AAGTCTGTGACAGGTTCAGGG	ATTGGCAAAAGCAACTGCGAG	502 bp

G

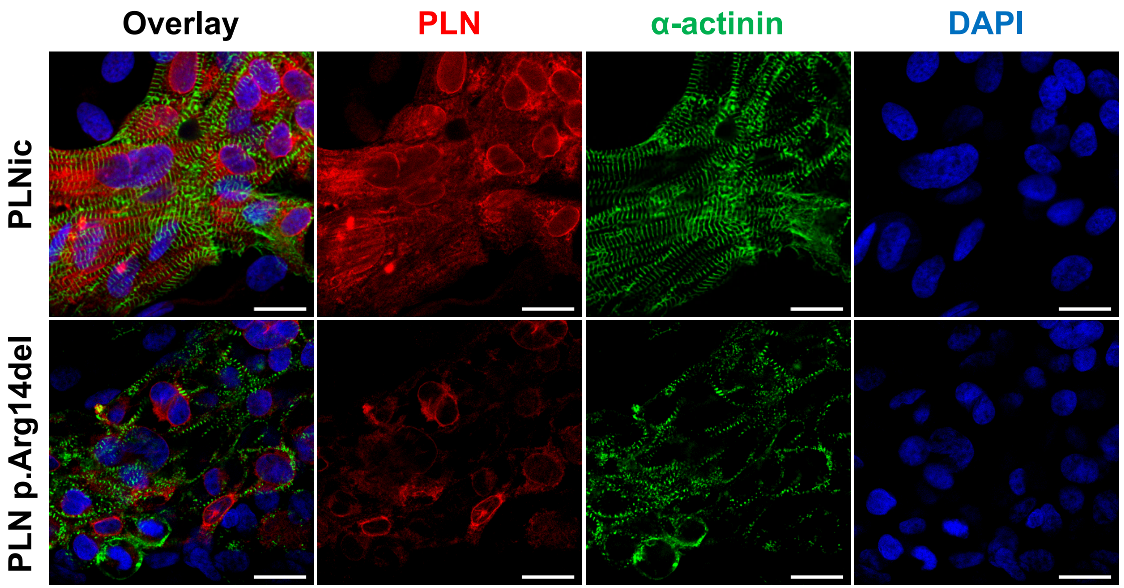

H

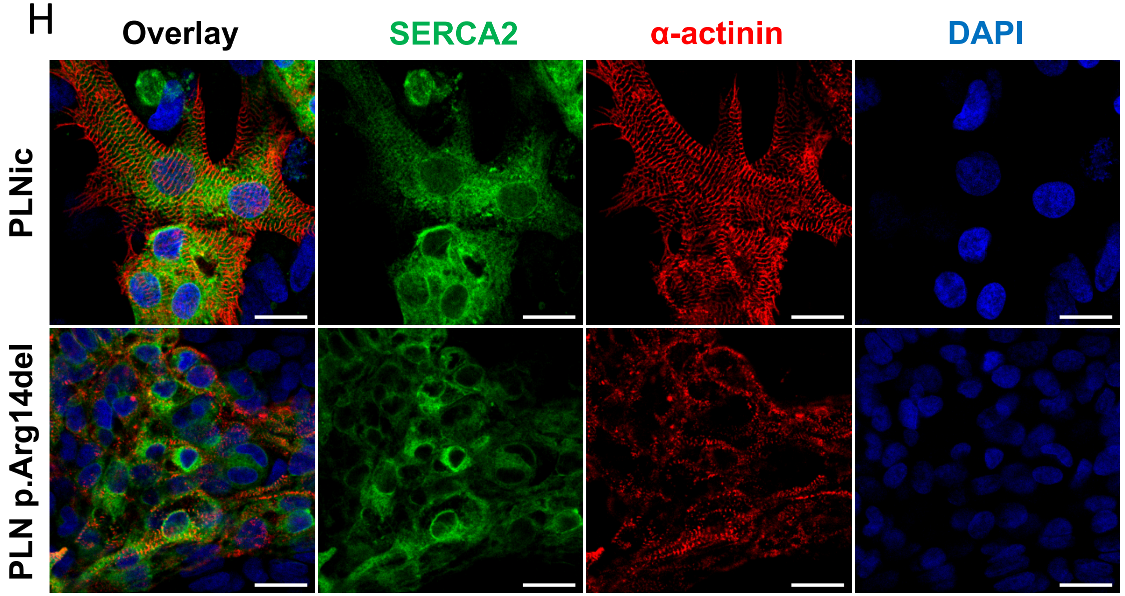

Appendix Figure S1. Characterization of hiPSC and hiPSC-CM. **A**: Bright field microscopy and immunofluorescence for pluripotency markers: **B:** Oct4 (red) and **C**: Tra-1-60 (green). G-banding chromosome analysis for **D:** PLN p.Arg14del hiPSC and **E:** isogenic control hiPSC demonstrating normal karyotype (46, XX). **F**: PCR primer sequences and estimated product size for off target (OT) sequencing. **G, H:** Immunofluorescence of 2D hiPSC-CM from PLNic and PLN p.Arg14del with antibodies against PLN (red), α-actinin (green and red), SERCA2 (green); DAPI staining for nuclei (blue); scale bar 20 µm.

Appendix Figure S2

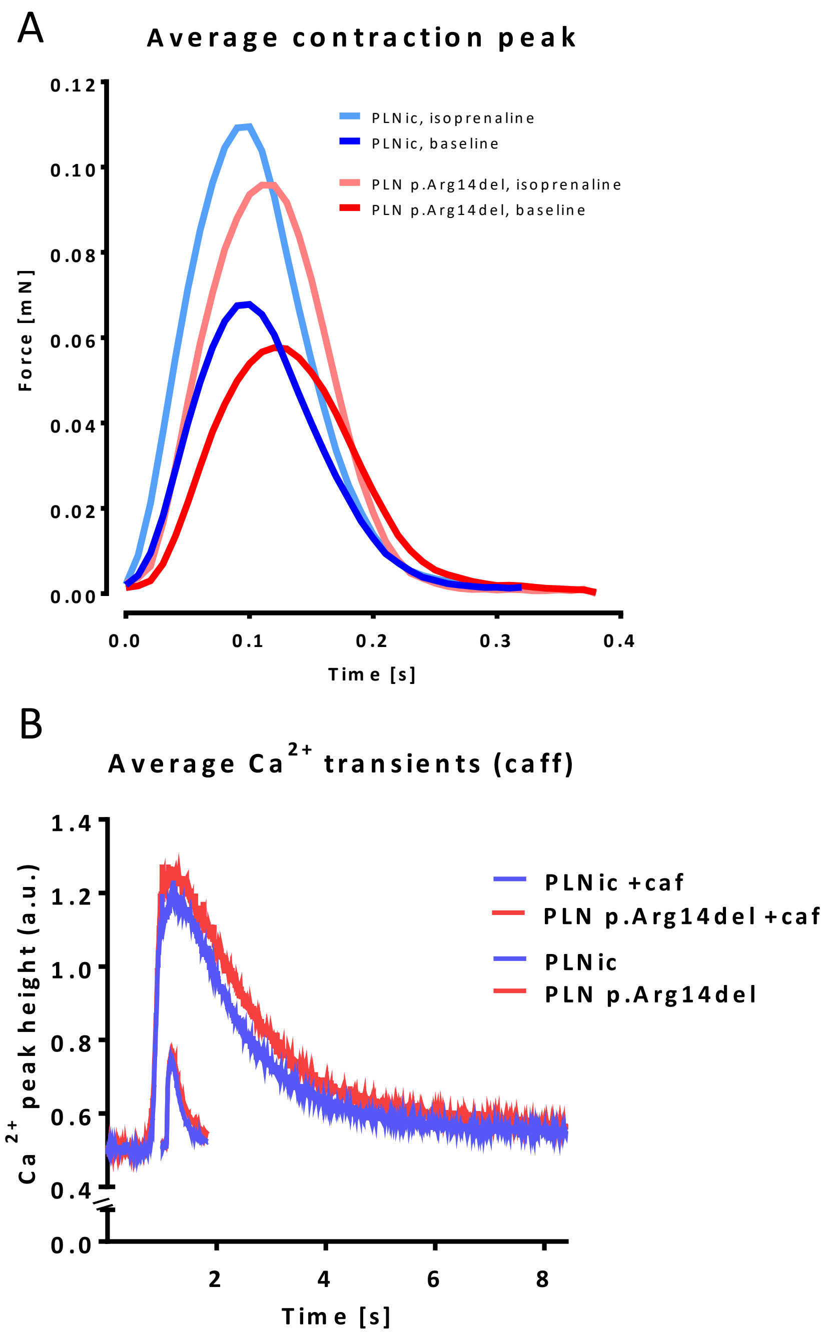

Appendix Figure S2. A: Average force contraction peak for PLNic and PLN p.Arg14del at baseline (EC50 [Ca^{2+}]) and in response to isoprenaline (ISO; 100 nM), data are related to Figure 2E, F. Ca^{2+} transient analysis in FURA 2-loaded 2D hiPSC-CM after caffeine puff (20 mM). **B:** Average Ca^{2+} transient peak, **C:** Ca^{2+} transient amplitude, **D:** Diastolic Ca^{2+}, **E:** Time to decay 80% and **F:** Ca^{2+} decay tau; PLNic: n=25 hiPSC-CM from 2 batches and PLN p.Arg14del: n=24 hiPSC-CM from 2 batches, mean ± SEM, Mann–Whitney U test, *$p<0.05$. The data are representative of n= 2 independent experiments.

Appendix Figure S3

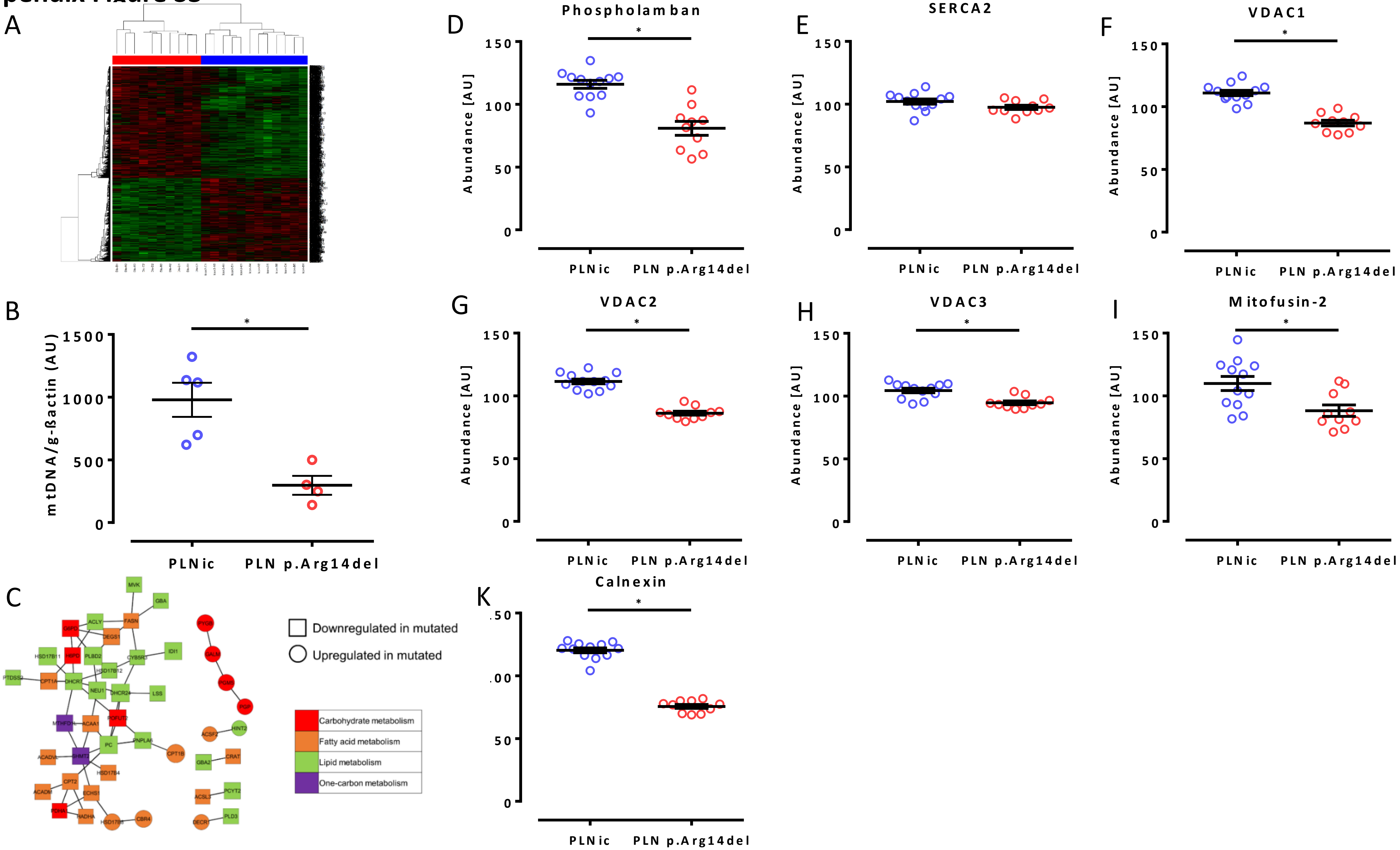

Appendix Figure S3. A: Heat map representation of differentially abundant proteins after unsupervised hierarchical clustering (ANOVA, FDR<0.05). **B:** PCR amplification of genomic DNA for the mitochondrial encoded NADH dehydrogenase (Mt-ND1/2), normalized to the nuclear-encoded gene actin; PLNic: n=5 EHTs, PLN p.Arg14del n=4 EHTs, mean±SEM, Mann–Whitney U test, *p< 0.05. **C:** Co-expression network of differentially abundant proteins, which are related to metabolism (according to Uniprot characterization). **D-K:** Protein abundance of selected candidate proteins in the proteome screen, data extracted from **Table EV1**, PLNic: n=12, PLN p.Arg14del n=10, mean ± SEM, false discovery rate, * p<0.05.

Appendix Figure S4

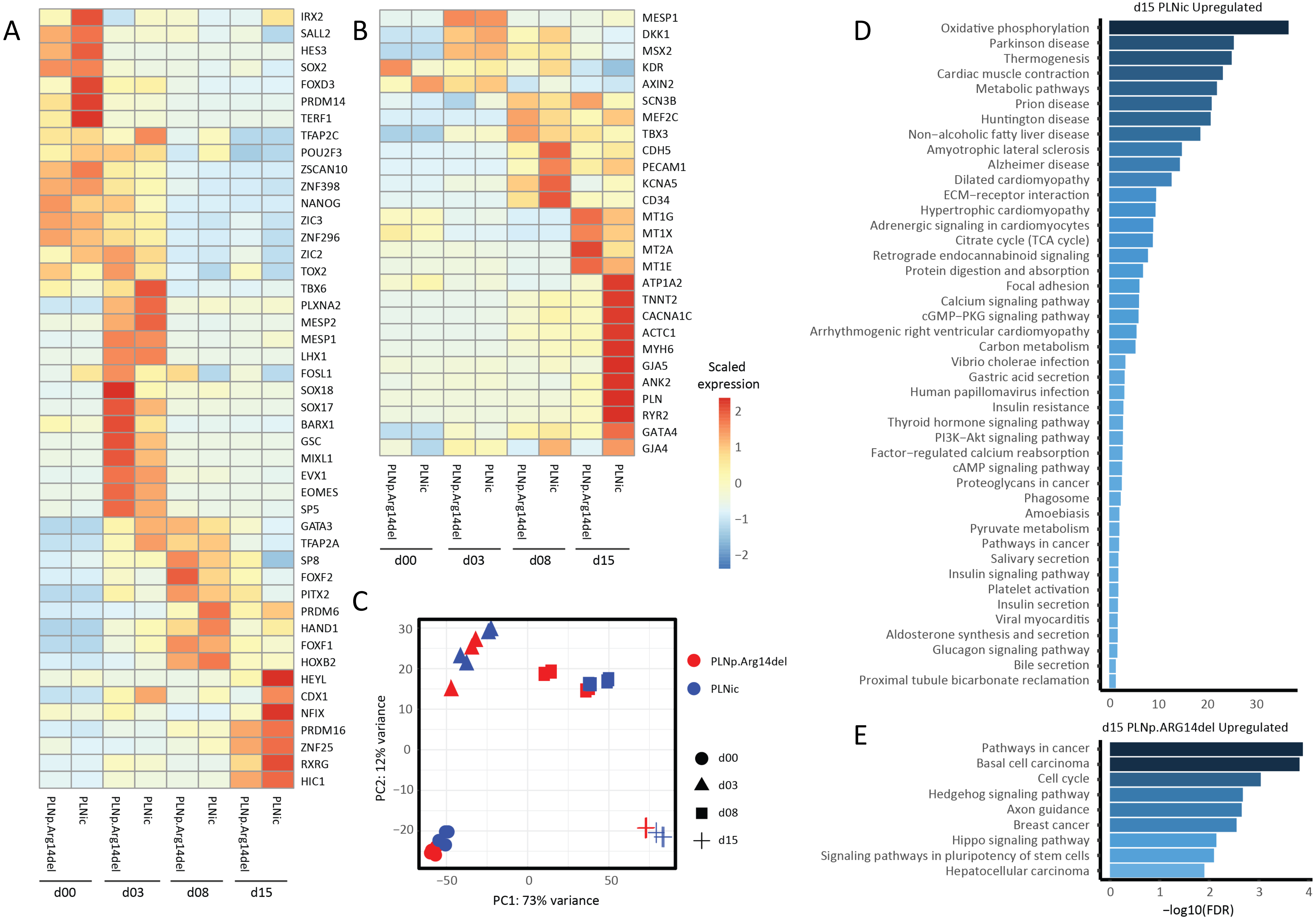

Appendix Figure S4. RNA sequencing analysis at different time points of cardiac differentiation of PLNic and PLN p.Arg14del hiPSC on day 0 (d00; hiPSC), day 3 (d03; mesodermal progenitors), day 8 (d08; cardiac progenitors) and day 15 (d15; early cardiomyocytes). **A, B** Heatmap of stage-specific markers of cardiac differentiation**.** The heatmap is scaled by row (gene) and the selected genes correctly define different stages of differentiation for both, PLNic and PLN p.Arg14del. **C:** Principal component analysis of PLNic and PLN p.Arg14del, n= 3-4 replicates, The data are representative of n= 2 independent differentiation rounds. **D, E:** Analysis of KEGG pathways of d15 samples that were significantly upregulated in PLNic (**D**) and PLN p.Arg14del (**E)**.

Appendix Figure S5

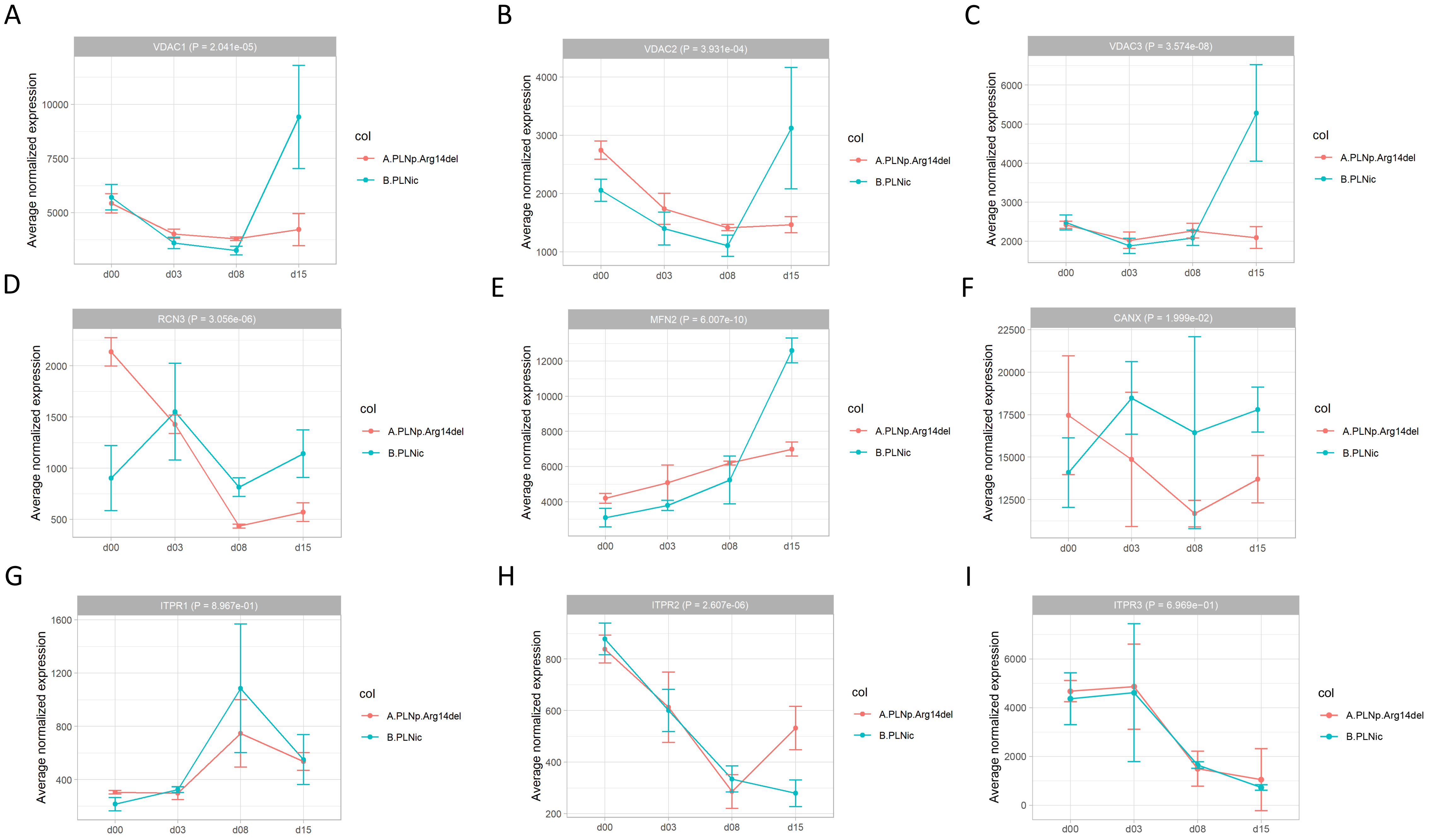

Appendix Figure S5. RNA sequencing analysis. **A-I:** Transcriptional expression profile of *VDAC1*, *VDAC2*, *VDAC3*, *MFN2*, *CANX, RCN3, ITPR1, ITPR2, ITPR3* at day 0, 3, 8, 15 of cardiac differentiation of PLNic and PLN p.Arg14del. 'P' indicates the adjusted p value of the difference in gene expression between PLNic and PLN p.Arg14del calculated by DESeq2 at day 15 of cardiac differentiation.

Appendix Figure S6

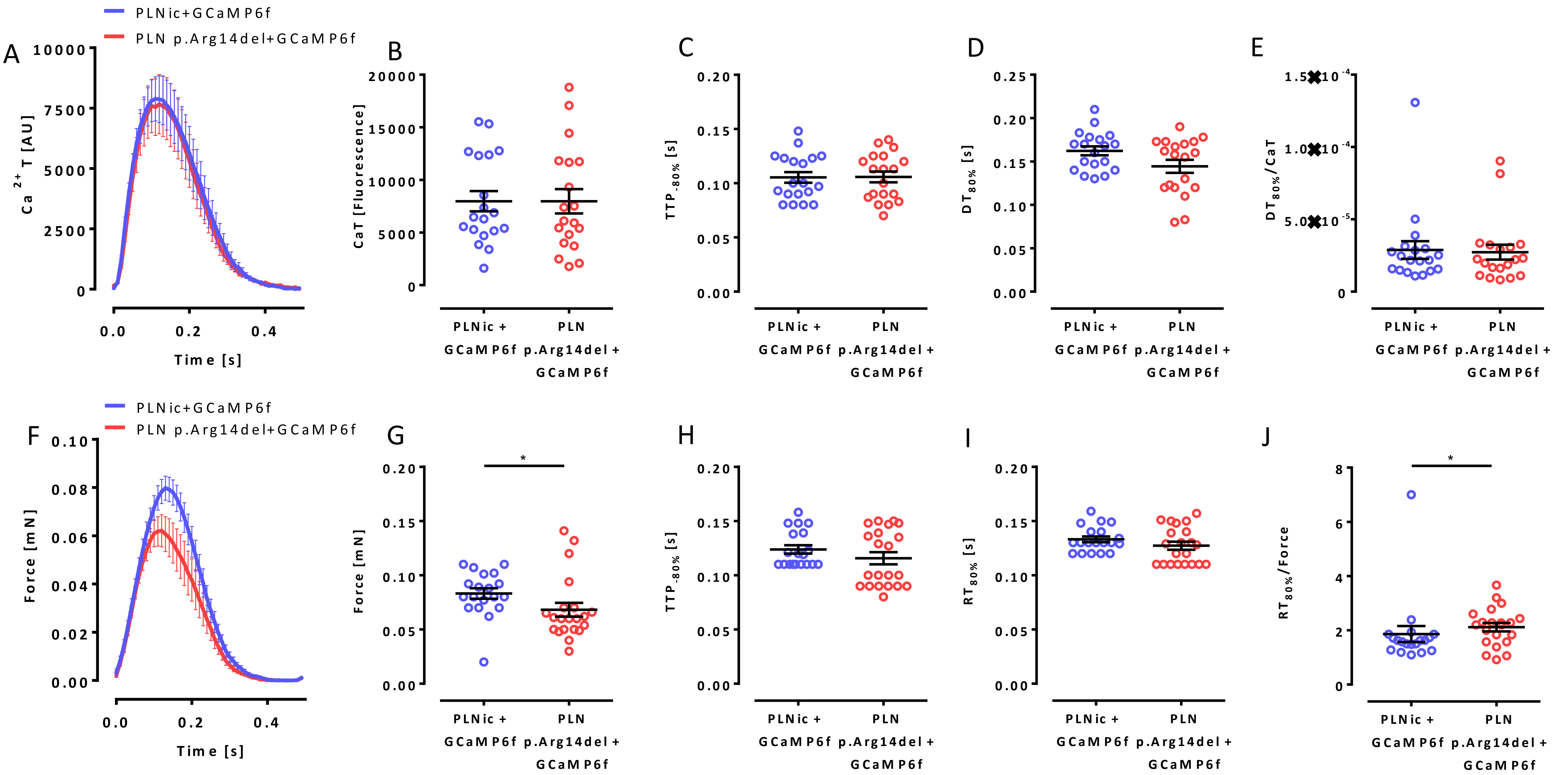

Appendix Figure S6. Ca^{2+} transient and force analysis of GCaMP6f-transduced EHTs. **A**: Average Ca^{2+} transient peak, **B:** Fluorescence amplitude, **C:** Ca^{2+} time to peak ($TTP_{80\%}$), **D:** Ca^{2+} decay time ($DT_{80\%}$), **E:** $DT_{80\%}$/fluorescence amplitude ratio, **F**: Average force peak, **G:** force amplitude, **H:** **f**orce time to peak ($TTP_{80\%}$), **I:** **f**orce relaxation time ($RT_{80\%}$), **J:** $RT_{80\%}$/force amplitude ratio. Mean ± SEM, PLNic: n=19 EHTs; PLN p.Arg14del: n=21 EHTs. Mann–Whitney U test, * $p<0.05$. The data are representative of 3 different batches.

Appendix Figure S7

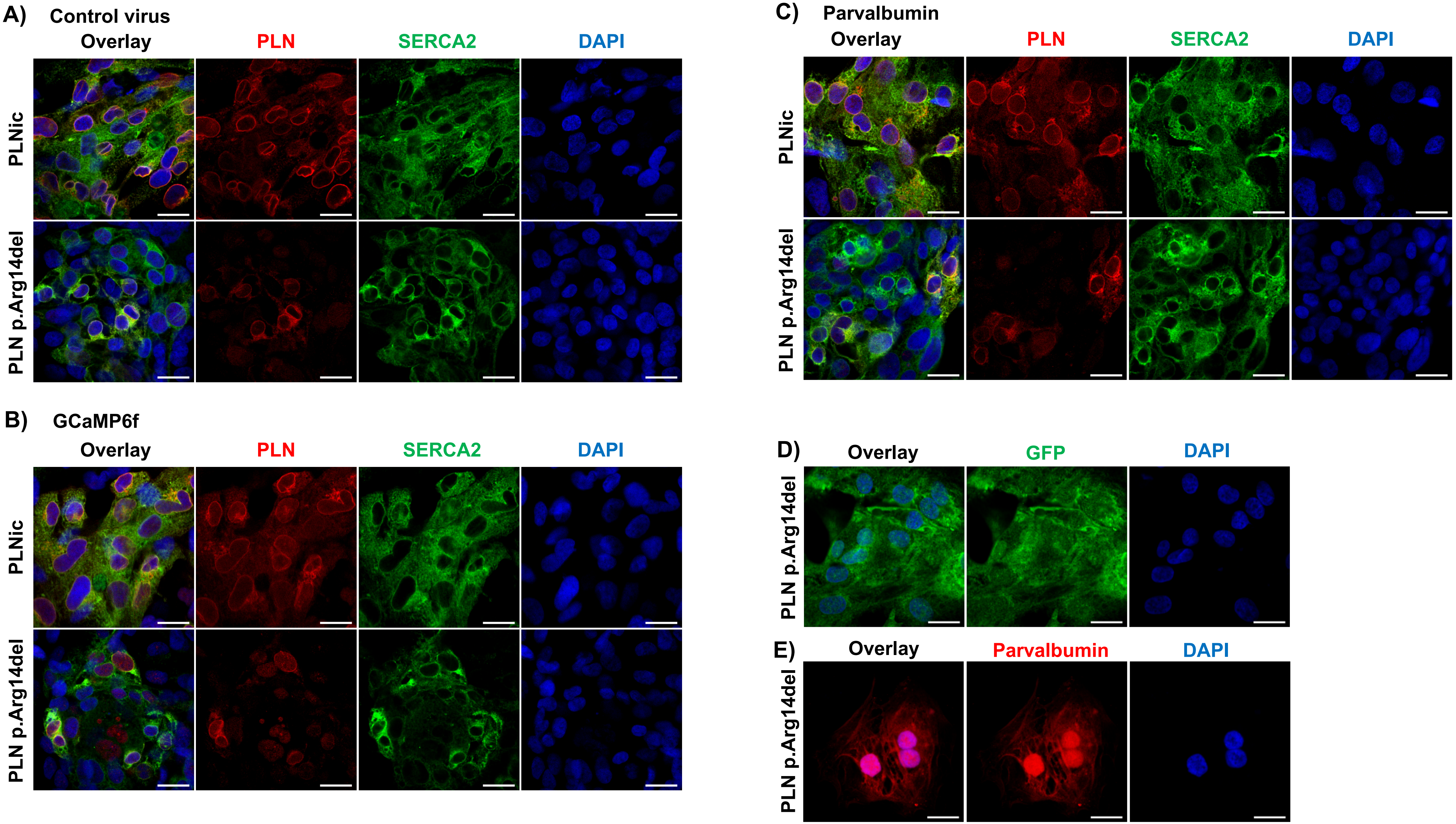

Appendix Figure S7. Immunofluorescence of 2D hiPSC-CM from PLNic and PLN p.Arg14del after transduction with a control virus **A.**, a virus encoding for GCaMP6f **B**. or parvalbumin **C**. with antibodies recognizing PLN (red), SERCA2 (green), DAPI staining for nuclei (blue), **D**. PLN p.Arg14del hiPSC-CM after transduction with a virus encoding for GCaMP6f using an antibody detecting GFP, **E**. PLN p.Arg14del hiPSC-CM after transduction with a virus encoding for parvalbumin with an antibody detecting parvalbumin; scale bar 20 µm.

Appendix Figure S8

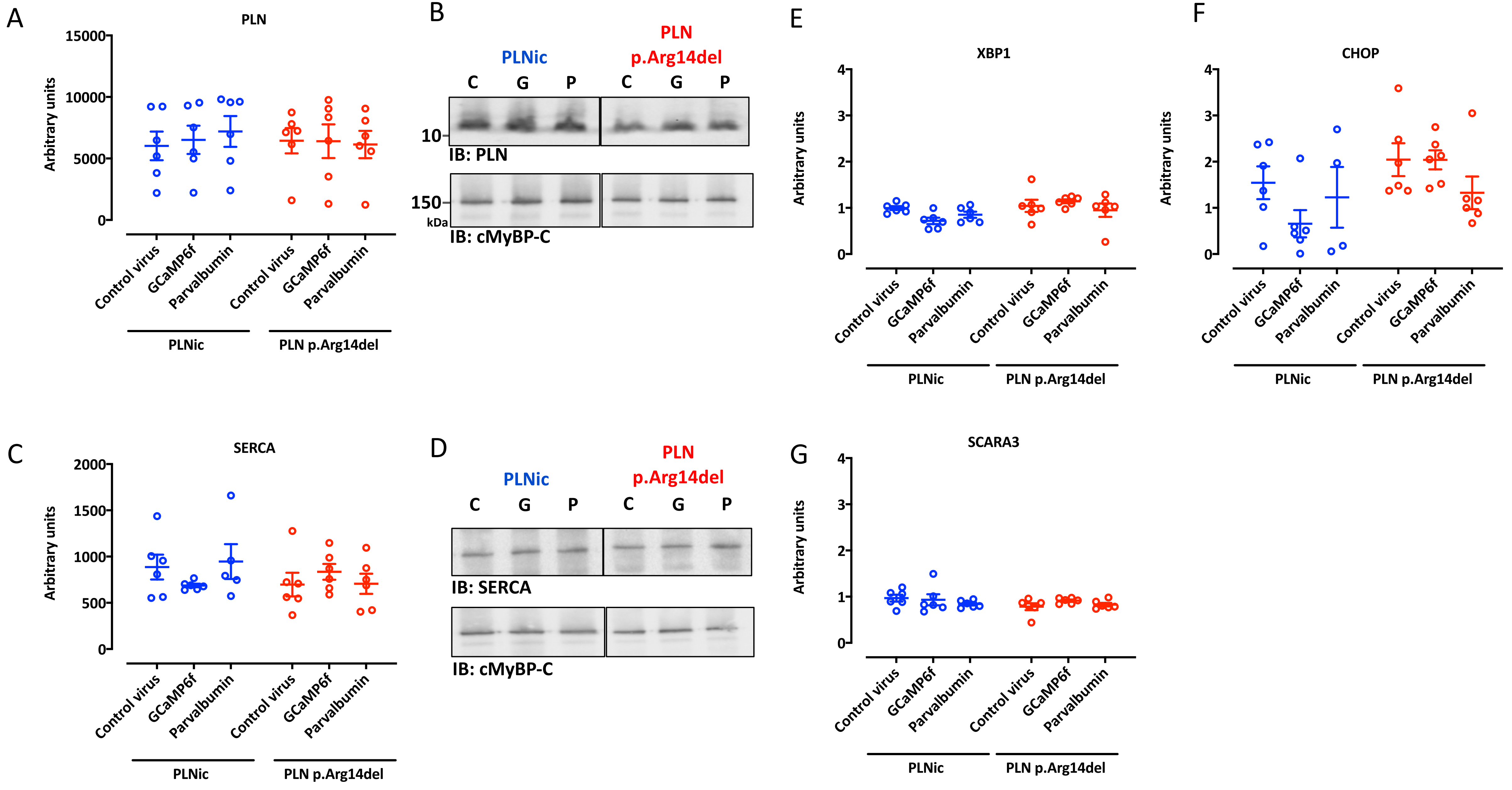

Appendix Figure S8. A-D. Western immunoblots for PLN and SERCA2 for PLNic and PLN p.Arg14del hiPSC-CM after transduction with control-, GCaMP6f- or parvalbumin-virus, n= 6-9, each replicate consists of a pool of 2-3 EHTs. Two-way ANOVA (comparing genotypes and virus conditions with Šidák`s post-test, mean ± SEM, *p<0.05. C: control virus, G: GCaMP6f virus. P: parvalbumin virus. **E-G.** Quantitative PCR of ER stress marker genes**.** PLNic and PLN p.Arg14del EHT after transduction with control-, GCaMP6f- or parvalbumin-virus; n= 6; each replicate consists of a pool of 2-3 EHTs. Two-way ANOVA (comparing genotypes and virus conditions) with Šidák`s post-test, mean ± SEM , *p<0.05.

Appendix Figure S9

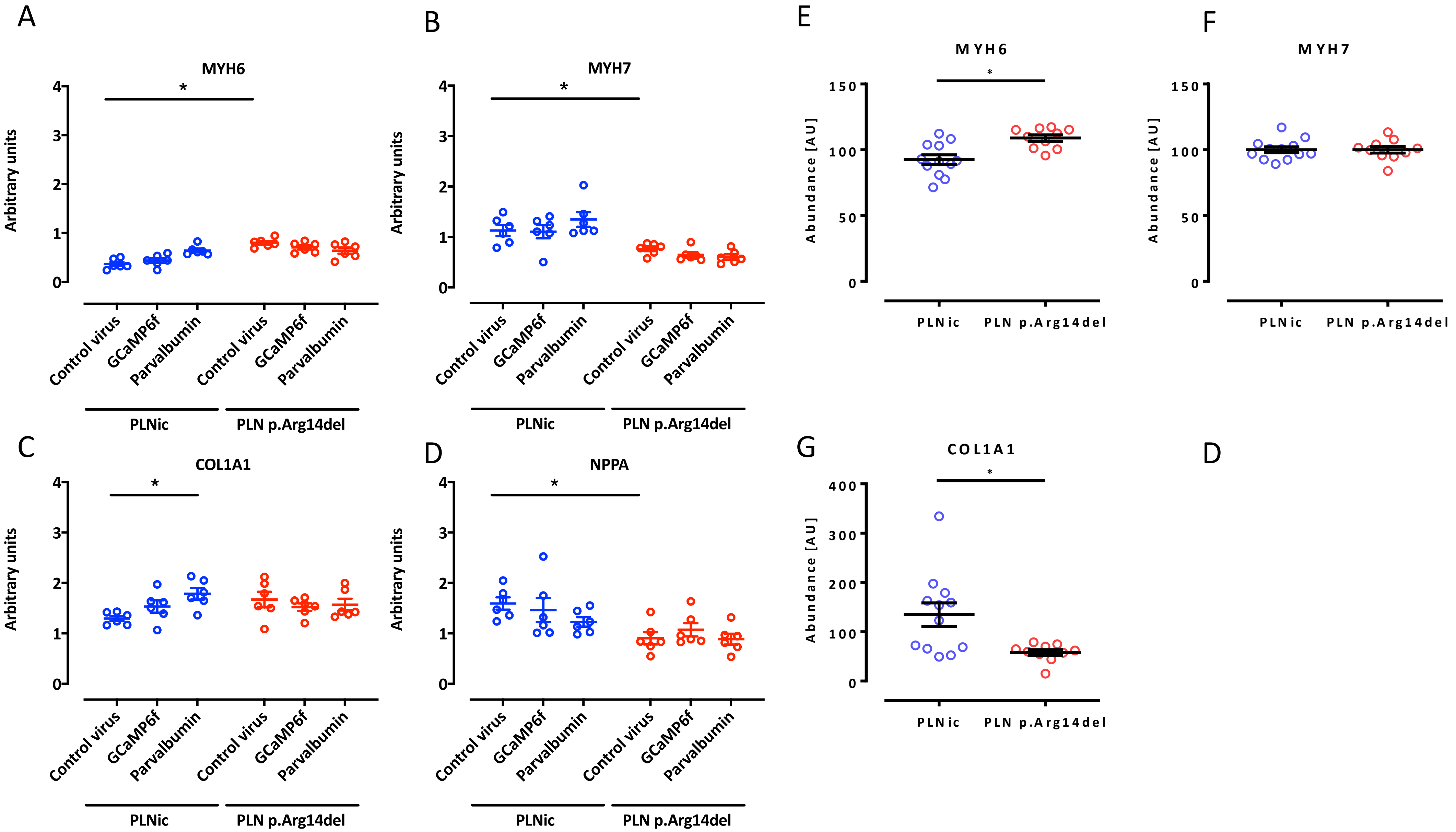

Appendix Figure S9. Fetal gene program markers. **A.** Quantitative PCR for *MYH6*, *MYH7*, *COL1A1* and *NPPA* of PLNic and PLN p.Arg14del EHTs after transduction with control- , GCaMP6f- or parvalbumin-virus; n= 6; each replicate consists of a pool of 2-3 EHTs. Two-way ANOVA (comparing genotypes and virus conditions) with Šidák`s post-test, mean ± SEM, *p<0.05. B. Protein abundance of MYH6, MYH7 and COL1A1 in the proteomic screen, data extracted from **Table EV1**, PLNic: n=12, PLN p.Arg14del n=10, mean±SEM, false discovery rate, * p<0.05. NPPA not detected.

Figure	Sample	Replicate number	p value	Statistical test
**2B**	NFH left ventricle	5	0,0002	1way ANOVA
	PLNic, EHT	8		
	PLN p.Arg14del, EHT	8		
**2D**	PLNic, EHT, baseline	8	0,0417	2way ANOVA
	PLNic, EHT, ISO	8	PM vs. PM + Iso	
	PLN p.Arg14del, EHT baseline	8		
	PLN p.Arg14del, EHT, ISO	8		
**2E**	PLNic, EHT, baseline	27, 6 experiments	< 0.0001	1way ANOVA
	PLNic, EHT, ISO	27, 6 experiments		
	PLN p.Arg14del, EHT baseline	19, 6 experiments	< 0.0001	1way ANOVA
	PLN p.Arg14del, EHT, ISO	19, 6 experiments		
**2F**	PLNic, EHT, baseline	27, 6 experiments	< 0.0001	1way ANOVA
	PLNic, EHT, ISO	27, 6 experiments		
	PLN p.Arg14del, EHT baseline	19, 6 experiments	< 0.0001	1way ANOVA
	PLN p.Arg14del, EHT, ISO	19, 6 experiments		
**3A**	PLNic, EHT	70, 10 experiments	< 0.0001	Mann-Whitney U
	PLN p.Arg14del, EHT	77, 10 experiments		
**3B**	PLNic, EHT	70, 10 experiments	< 0.0001	Mann-Whitney U
	PLN p.Arg14del, EHT	77, 10 experiments		
**3C**	PLNic, EHT	67, 9 experiments	< 0.0001	Mann-Whitney U
	PLN p.Arg14del, EHT	65, 9 experiments		
**3D**	PLNic, EHT	67, 9 experiments	0,0106	Mann-Whitney U
	PLN p.Arg14del, EHT	65, 9 experiments		
**3G**	PLNic, EHT	21, 8 experiments	< 0.0001	Unpaired two-sided Student's t-test
	PLN p.Arg14del, EHT	35, 8 experiments		
**3H**	PLNic, EHT	67, 9 experiments	0,1287	Unpaired two-sided Student's t-test
	PLN p.Arg14del, EHT	65, 9 experiments		
**3I**	PLNic, EHT	67, 9 experiments	0,1604	Unpaired two-sided Student's t-test
	PLN p.Arg14del, EHT	65, 9 experiments		
**3J**	PLNic, EHT	67, 9 experiments	< 0.0001	Unpaired two-sided Student's t-test
	PLN p.Arg14del, EHT	65, 9 experiments		
**4B**	PLNic, 2D hiPSC-CM	25, 2 experiments	0,6519	Mann-Whitney U
	PLN p.Arg14del, 2D hiPSC-CM	24, 2 experiments		
**4C**	PLNic, 2D hiPSC-CM	25, 2 experiments	0,8313	Mann-Whitney U
	PLN p.Arg14del, 2D hiPSC-CM	24, 2 experiments		
**4D**	PLNic, 2D hiPSC-CM	25, 2 experiments	0,0011	Mann-Whitney U
	PLN p.Arg14del, 2D hiPSC-CM	24, 2 experiments		
**4E**	PLNic, 2D hiPSC-CM	25, 2 experiments	0,0646	Mann-Whitney U
	PLN p.Arg14del, 2D hiPSC-CM	24, 2 experiments		
**4G**	PLNic, EHT	24, 32, 41, 5 experiments	0.6537 (interaction)	2way ANOVA, Tukey's post test
	PLN p.Arg14del, EHT	15, 24, 23, 5 experiments	0.0187 (row factor)	
			< 0,0001 (column factor)	
**5C**	PLNic, EHT	12, 2 batches	< 0.05	Benjamini-Hochberg, Multiple testing
	PLN p.Arg14del, EHT	10, 1 batch		

Figure	Sample	Replicate number	p value	Statistical test
**6B**	PLNic, 2D hiPSC-CM	6	0,0022	Mann-Whitney U
	PLN p.Arg14del, 2D hiPSC-CM	6		
**6C**	PLNic, 2D hiPSC-CM	6	0,0152	Mann-Whitney U
	PLN p.Arg14del, 2D hiPSC-CM	6		
**6D**	PLNic, 2D hiPSC-CM	6	0,0022	Mann-Whitney U
	PLN p.Arg14del, 2D hiPSC-CM	6		
**6E**	PLNic, EHT	8	0,0002	Mann-Whitney U
	PLN p.Arg14del, EHT	8		
**7B**	NFH	3, 15	< 0.0001	1way ANOVA, Tukey's post test
	FH ICM	2, 10		
	FH PLN CM	3, 14		
**7C**	NFH	3, 15	< 0.0001	1way ANOVA, Tukey's post test
	FH ICM	3, 15		
	FH PLN CM	3, 15		
**8A**	PLNic, EHT, control virus	6	0.0005 (interaction)	2way ANOVA, Sidak's post test
	PLNic, EHT, GCaMP6f	6	0.0007 (row factor)	
	PLNic, EHT, parvalbumin	6	0.0001 (column factor)	
	PLN p.Arg14del, EHT, control virus	6		
	PLN p.Arg14del, EHT, GCaMP6f	6		
	PLN p.Arg14del, EHT, parvalbumin	6		
**8C**	PLNic, EHT, control virus	9	0.6280 (interaction)	2way ANOVA, Sidak's post test
	PLNic, EHT, GCaMP6f	9	0.0186 (row factor)	
	PLNic, EHT, parvalbumin	9	< 0.0001 (column factor)	
	PLN p.Arg14del, EHT, control virus	9		
	PLN p.Arg14del, EHT, GCaMP6f	9		
	PLN p.Arg14del, EHT, parvalbumin	9		
**8E**	PLNic, EHT, control virus	24	0.0001 (interaction)	2way ANOVA, Sidak's post test
	PLNic, EHT, GCaMP6f	23	0.0003 (row factor)	
	PLNic, EHT, parvalbumin	24	< 0.0001 (column factor)	
	PLN p.Arg14del, EHT, control virus	22		
	PLN p.Arg14del, EHT, GCaMP6f	24		
	PLN p.Arg14del, EHT, parvalbumin	22		
**8F**	PLNic, EHT, control virus	6	0.0002 (interaction)	2way ANOVA, Sidak's post test
	PLNic, EHT, GCaMP6f	6	0.0664 (row factor)	
	PLNic, EHT, parvalbumin	6	0.0010 (column factor)	
	PLN p.Arg14del, EHT, control virus	6		
	PLN p.Arg14del, EHT, GCaMP6f	6		
	PLN p.Arg14del, EHT, parvalbumin	6		
**8G**	PLNic, EHT, control virus	6	0.0007 (interaction)	2way ANOVA, Sidak's post test
	PLNic, EHT, GCaMP6f	6	< 0.0001 (row factor)	
	PLNic, EHT, parvalbumin	6	< 0.0001 (column factor)	
	PLN p.Arg14del, EHT, control virus	6		
	PLN p.Arg14del, EHT, GCaMP6f	6		
	PLN p.Arg14del, EHT, parvalbumin	6		
**8H**	PLNic, EHT, control virus	6	0.0693 (interaction)	2way ANOVA, Sidak's post test
	PLNic, EHT, GCaMP6f	6	0.0387 (row factor)	
	PLNic, EHT, parvalbumin	6	0.8969 (column factor)	
	PLN p.Arg14del, EHT, control virus	6		
	PLN p.Arg14del, EHT, GCaMP6f	6		
	PLN p.Arg14del, EHT, parvalbumin	6		

Figure	Sample	Replicate number	p value	Statistical test
**6B**	PLNic, 2D hiPSC-CM	6	0,0022	Mann-Whitney U
	PLN p.Arg14del, 2D hiPSC-CM	6		
**6C**	PLNic, 2D hiPSC-CM	6	0,0152	Mann-Whitney U
	PLN p.Arg14del, 2D hiPSC-CM	6		
**6D**	PLNic, 2D hiPSC-CM	6	0,0022	Mann-Whitney U
	PLN p.Arg14del, 2D hiPSC-CM	6		
**6E**	PLNic, EHT	8	0,0002	Mann-Whitney U
	PLN p.Arg14del, EHT	8		
**7B**	NFH	3, 15	< 0.0001	1way ANOVA, Tukey's post test
	FH ICM	2, 10		
	FH PLN CM	3, 14		
**7C**	NFH	3, 15	< 0.0001	1way ANOVA, Tukey's post test
	FH ICM	3, 15		
	FH PLN CM	3, 15		
**8A**	PLNic, EHT, control virus	6	0.0005 (interaction)	2way ANOVA, Sidak's post test
	PLNic, EHT, GCaMP6f	6	0.0007 (row factor)	
	PLNic, EHT, parvalbumin	6	0.0001 (column factor)	
	PLN p.Arg14del, EHT, control virus	6		
	PLN p.Arg14del, EHT, GCaMP6f	6		
	PLN p.Arg14del, EHT, parvalbumin	6		
**8C**	PLNic, EHT, control virus	9	0.6280 (interaction)	2way ANOVA, Sidak's post test
	PLNic, EHT, GCaMP6f	9	0.0186 (row factor)	
	PLNic, EHT, parvalbumin	9	< 0.0001 (column factor)	
	PLN p.Arg14del, EHT, control virus	9		
	PLN p.Arg14del, EHT, GCaMP6f	9		
	PLN p.Arg14del, EHT, parvalbumin	9		
**8E**	PLNic, EHT, control virus	24	0.0001 (interaction)	2way ANOVA, Sidak's post test
	PLNic, EHT, GCaMP6f	23	0.0003 (row factor)	
	PLNic, EHT, parvalbumin	24	< 0.0001 (column factor)	
	PLN p.Arg14del, EHT, control virus	22		
	PLN p.Arg14del, EHT, GCaMP6f	24		
	PLN p.Arg14del, EHT, parvalbumin	22		
**8F**	PLNic, EHT, control virus	6	0.0002 (interaction)	2way ANOVA, Sidak's post test
	PLNic, EHT, GCaMP6f	6	0.0664 (row factor)	
	PLNic, EHT, parvalbumin	6	0.0010 (column factor)	
	PLN p.Arg14del, EHT, control virus	6		
	PLN p.Arg14del, EHT, GCaMP6f	6		
	PLN p.Arg14del, EHT, parvalbumin	6		
**8G**	PLNic, EHT, control virus	6	0.0007 (interaction)	2way ANOVA, Sidak's post test
	PLNic, EHT, GCaMP6f	6	< 0.0001 (row factor)	
	PLNic, EHT, parvalbumin	6	< 0.0001 (column factor)	
	PLN p.Arg14del, EHT, control virus	6		
	PLN p.Arg14del, EHT, GCaMP6f	6		
	PLN p.Arg14del, EHT, parvalbumin	6		
**8H**	PLNic, EHT, control virus	6	0.0693 (interaction)	2way ANOVA, Sidak's post test
	PLNic, EHT, GCaMP6f	6	0.0387 (row factor)	
	PLNic, EHT, parvalbumin	6	0.8969 (column factor)	
	PLN p.Arg14del, EHT, control virus	6		
	PLN p.Arg14del, EHT, GCaMP6f	6		
	PLN p.Arg14del, EHT, parvalbumin	6		

Figure	Sample	Replicate number	p value	Statistical test
**EV1B**	PLNic, EHT, pPDH	6	0,0013	Unpaired two-sided Student's t-test
	PLN p.Arg14del, EHT, pPDH	6		
**EV1B**	PLNic, EHT, pAMPK	6	0,0072	Unpaired two-sided Student's t-test
	PLN p.Arg14del, EHT, pAMPK	6		
**EV1B**	PLNic, EHT, galectin-3	3	0,0252	Unpaired two-sided Student's t-test
	PLN p.Arg14del, EHT, galectin-3	3		
**EV1B**	PLNic, EHT, SLTM	3	0,0006	Unpaired two-sided Student's t-test
	PLN p.Arg14del, EHT, SLTM	3		
**EV1B**	PLNic, EHT, LMCD1	3	0,0006	Unpaired two-sided Student's t-test
	PLN p.Arg14del, EHT, LMCD1	3		
**EV1B**	PLNic, EHT, calnexin	6	0,0009	Unpaired two-sided Student's t-test
	PLN p.Arg14del, EHT, calnexin	6		
**EV1B**	PLNic, EHT, reticulocalbin-3	3	0,1592	Unpaired two-sided Student's t-test
	PLN p.Arg14del, EHT, reticulocalbin-3	3		
**Appendix Figure S2 C**	PLNic, 2D hiPSC-CM	25, 2 experiments	0,1158	Mann-Whitney U
	PLN p.Arg14del, 2D hiPSC-CM	24, 2 experiments		
**Appendix Figure S2 D**	PLNic, 2D hiPSC-CM	25, 2 experiments	0,7249	Mann-Whitney U
	PLN p.Arg14del, 2D hiPSC-CM	24, 2 experiments		
**Appendix Figure S2 E**	PLNic, 2D hiPSC-CM	25, 2 experiments	0,2926	Mann-Whitney U
	PLN p.Arg14del, 2D hiPSC-CM	24, 2 experiments		
**Appendix Figure S2 F**	PLNic, 2D hiPSC-CM	25, 2 experiments	0,5632	Mann-Whitney U
	PLN p.Arg14del, 2D hiPSC-CM	24, 2 experiments		
**Appendix Figure S3B**	PLNic, EHT	5	0,0159	Unpaired two-sided Student's t-test
	PLN p.Arg14del, EHT	4		
**Appendix Figure S3D-K**	PLNic, EHT	12, 2 batches	< 0.05	Benjamini-Hochberg, Multiple testi
	PLN p.Arg14del, EHT	10, 1 batch		
**Appendix Figure S5A**	PLNic, hiPSC EBs	3-4/time point	$2.04x10^{-5}$	Unpaired two-sided Student's t-test
	PLN p.Arg14del, hiPSC EBs	3-4/time point		
**Appendix Figure S5B**	PLNic, hiPSC EBs	3-4/time point	$3.031x10^{-4}$	Unpaired two-sided Student's t-test
	PLN p.Arg14del, hiPSC EBs	3-4/time point		
**Appendix Figure S5C**	PLNic, hiPSC EBs	3-4/time point	$3.574x10^{-8}$	Unpaired two-sided Student's t-test
	PLN p.Arg14del, hiPSC EBs	3-4/time point		
**Appendix Figure S5D**	PLNic, hiPSC EBs	3-4/time point	$3.056x10^{-6}$	Unpaired two-sided Student's t-test
	PLN p.Arg14del, hiPSC EBs	3-4/time point		
**Appendix Figure S5E**	PLNic, hiPSC EBs	3-4/time point	$6.007x10^{-10}$	Unpaired two-sided Student's t-test
	PLN p.Arg14del, hiPSC EBs	3-4/time point		
**Appendix Figure S5F**	PLNic, hiPSC EBs	3-4/time point	$1.999x10^{-2}$	Unpaired two-sided Student's t-test
	PLN p.Arg14del, hiPSC EBs	3-4/time point		
**Appendix Figure S5G**	PLNic, hiPSC EBs	3-4/time point	$8.967x10^{-1}$	Unpaired two-sided Student's t-test
	PLN p.Arg14del, hiPSC EBs	3-4/time point		
**Appendix Figure S5H**	PLNic, hiPSC EBs	3-4/time point	$2.607x10^{-6}$	Unpaired two-sided Student's t-test
	PLN p.Arg14del, hiPSC EBs	3-4/time point		
**Appendix Figure S5I**	PLNic, hiPSC EBs	3-4/time point	$6.969x10^{-1}$	Unpaired two-sided Student's t-test
	PLN p.Arg14del, hiPSC EBs	3-4/time point		

Figure	Sample	Replicate number	p value	Statistical test
**Appendix Figure S6B**	PLNic, EHT	19	0,8326	Mann-Whitney U
	PLN p.Arg14del, EHT	21		
**Appendix Figure S6C**	PLNic, EHT	19	0,9827	Mann-Whitney U
	PLN p.Arg14del, EHT	21		
**Appendix Figure S6D**	PLNic, EHT	19	0,1069	Mann-Whitney U
	PLN p.Arg14del, EHT	21		
**Appendix Figure S6E**	PLNic, EHT	19	0,8326	Mann-Whitney U
	PLN p.Arg14del, EHT	21		
**Appendix Figure S6G**	PLNic, EHT	19	0,004	Mann-Whitney U
	PLN p.Arg14del, EHT	21		
**Appendix Figure S6H**	PLNic, EHT	19	0,1849	Mann-Whitney U
	PLN p.Arg14del, EHT	21		
**Appendix Figure S6I**	PLNic, EHT	19	0,154	Mann-Whitney U
	PLN p.Arg14del, EHT	21		
**Appendix Figure S6J**	PLNic, EHT	19	0,0379	Mann-Whitney U
	PLN p.Arg14del, EHT	21		
**Appendix Figure S8A**	PLNic, EHT	6	0.8190 (interaction)	2way ANOVA, Sidak's post test
	PLN p.Arg14del, EHT	6	0.9350 (row factor)	
			0.8009 (column factor)	
**Appendix Figure S8C**	PLNic, EHT	6	0.2136 (interaction)	2way ANOVA, Sidak's post test
	PLN p.Arg14del, EHT	6	0.8621 (row factor)	
			0.3368 (column factor)	
**Appendix Figure S8E**	PLNic, EHT	6	0.1022 (interaction)	2way ANOVA, Sidak's post test
	PLN p.Arg14del, EHT	6	0.4141 (row factor)	
			0.0168 (column factor)	
**Appendix Figure S8F**	PLNic, EHT	6	0.2124 (interaction)	2way ANOVA, Sidak's post test
	PLN p.Arg14del, EHT	6	0.3100 (row factor)	
			0.0338 (column factor)	
**Appendix Figure S8G**	PLNic, EHT	6	0.3794 (interaction)	2way ANOVA, Sidak's post test
	PLN p.Arg14del, EHT	6	0.4426 (row factor)	
			0.2064 (column factor)	
**Appendix Figure S9A**	PLNic, EHT	6	0.0002 (interaction)	2way ANOVA, Sidak's post test
	PLN p.Arg14del, EHT	6	0.2981 (row factor)	
			<0.0001 (column factor)	
**Appendix Figure S9B**	PLNic, EHT	6	0.1527 (interaction)	2way ANOVA, Sidak's post test
	PLN p.Arg14del, EHT	6	0.5926 (row factor)	
			<0.0001 (column factor)	
**Appendix Figure S9C**	PLNic, EHT	6	0.0374 (interaction)	2way ANOVA, Sidak's post test
	PLN p.Arg14del, EHT	6	0.1990 (row factor)	
			0.5857 (column factor)	
**Appendix Figure S9D**	PLNic, EHT	6	0.4352 (interaction)	2way ANOVA, Sidak's post test
	PLN p.Arg14del, EHT	6	0.2850 (row factor)	
			0.0003 (column factor)	
**Appendix Figure S9E**	PLNic, EHT	12, 2 batches	<0.05	Benjamini-Hochberg, Multiple testi
	PLN p.Arg14del, EHT	10, 1 batch		
**Appendix Figure S9F**	PLNic, EHT	12, 2 batches	<0.05	Benjamini-Hochberg, Multiple testi
	PLN p.Arg14del, EHT	10, 1 batch		
**Appendix Figure S9G**	PLNic, EHT	12, 2 batches	<0.05	Benjamini-Hochberg, Multiple testi
	PLN p.Arg14del, EHT	10, 1 batch		

Appendix Figure S10. Statistical details
