# Supplementary material for: Impairment of the ER/mitochondria compartment in human cardiomyocytes with PLN p.Arg14del mutation
Source: EMBO Mol Med. 2021 May 16;13(6):e13074. doi: 10.15252/emmm.202013074 (PMC8185541; doi:10.15252/emmm.202013074)
Supplement: Supplementary file 2 — Expanded View Figures PDF [file EMMM-13-e13074-s003.pdf]

Expanded View Figures

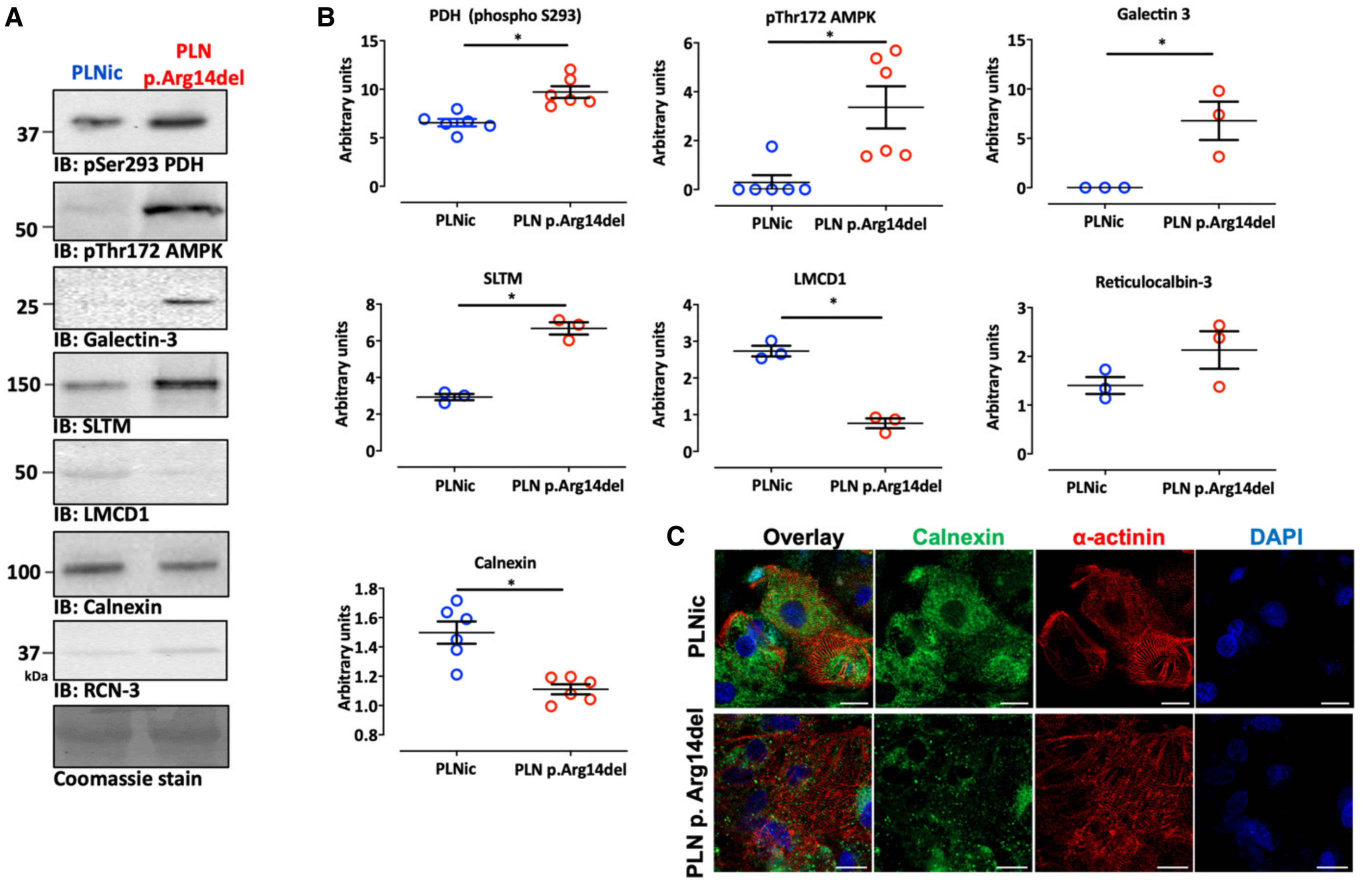

Figure EV1. Investigation of protein expression and localization.

A, B Western immunoblots for phosphorylated pyruvate dehydrogenase at pS293 (PDH; $n = 6$, each replicate consists of a pool of 3–4 EHTs from 2 different separate batches), AMP-dependent protein kinase at Thr172 (AMPK; $n = 6$, each replicate consists of a pool of 3–4 EHTs from 2 different separate batches), galectin-3 ($n = 3$; each replicate consists of a pool of 3–4 EHTs from 2 different separate batches), SAFB-like transcription modulator (SLTM; $n = 3$; each replicate consists of a pool of 3–4 EHTs from 2 different separate batches), LIM and cysteine-rich domains 1 (LMCD1; $n = 3$; each replicate consists of a pool of 3–4 EHTs from 2 different separate batches), calnexin ($n = 6$; each replicate consists of a pool of 3–4 EHTs from 2 different separate batches), and reticulocalbin-3 (RCN-3; $n = 3$; each replicate consists of a pool of 3–4 EHTs from 2 different separate batches); Coomassie staining was used as a loading control, mean ± SEM, unpaired two-sided Student's *t*-test, $*P < 0.05$.

C Immunofluorescence of 2D hiPSC-CM from PLNic and PLN p.Arg14del with antibodies recognizing calnexin (green), α-actinin (red), and DAPI staining for nuclei (blue); scale bar 20 μm.

Source data are available online for this figure.

 © 2021 The Authors

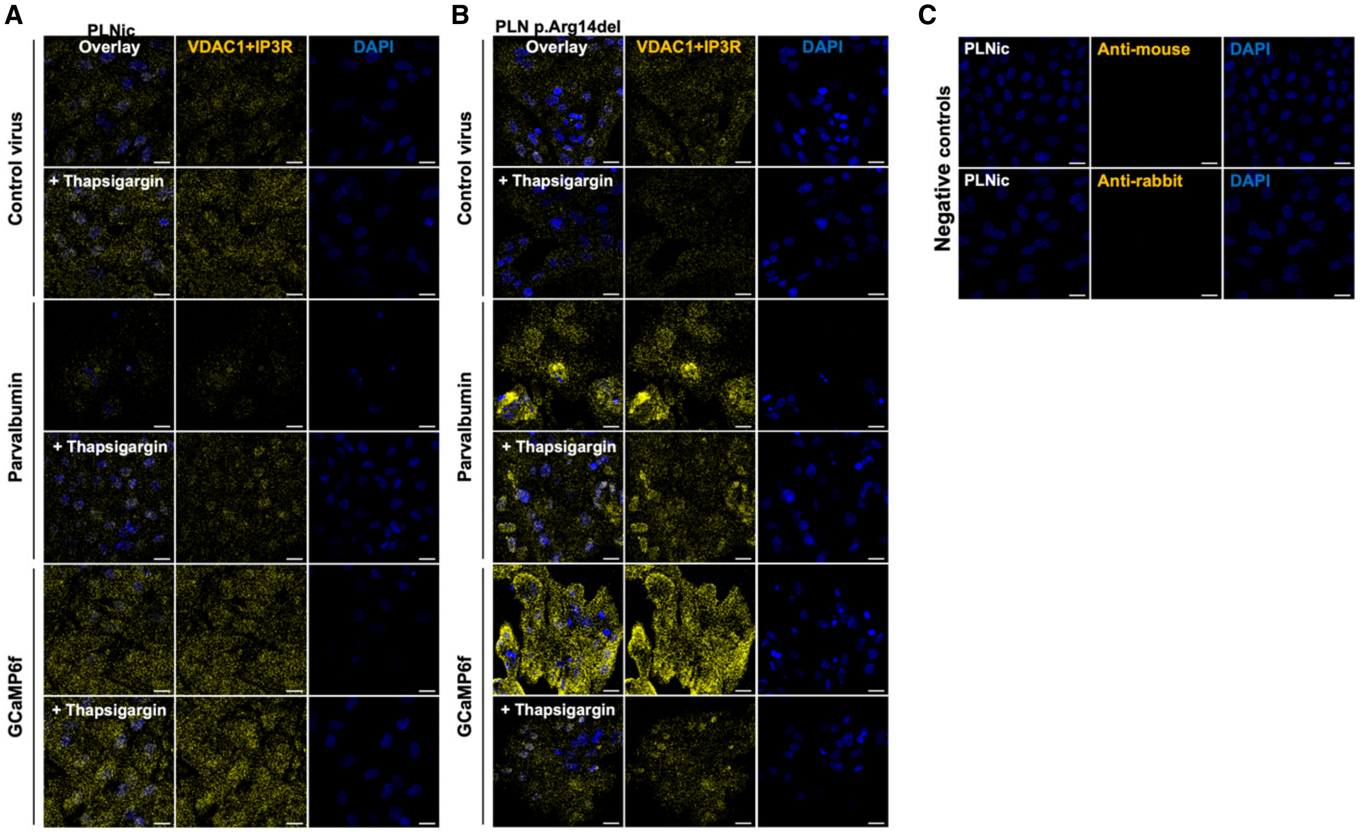

Figure EV2. Investigation of protein–protein interaction.

A, B Proximity ligation assays (PLAs; yellow) combining an anti-VDAC1 (localizing to the outer mitochondrial membrane) and an IP_3R-antibody (localizing to the ER) and DAPI staining of nuclei (blue) at baseline and after thapsigargin (1.0 μM, 5 h) exposure for PLNic (A) and PLN p.Arg14del (B) after transduction with control, GCaMP6f, or parvalbumin virus.

C Negative control: Anti-mouse, anti-rabbit antibodies alone. Scale bar 20 μm.

© 2021 The Authors

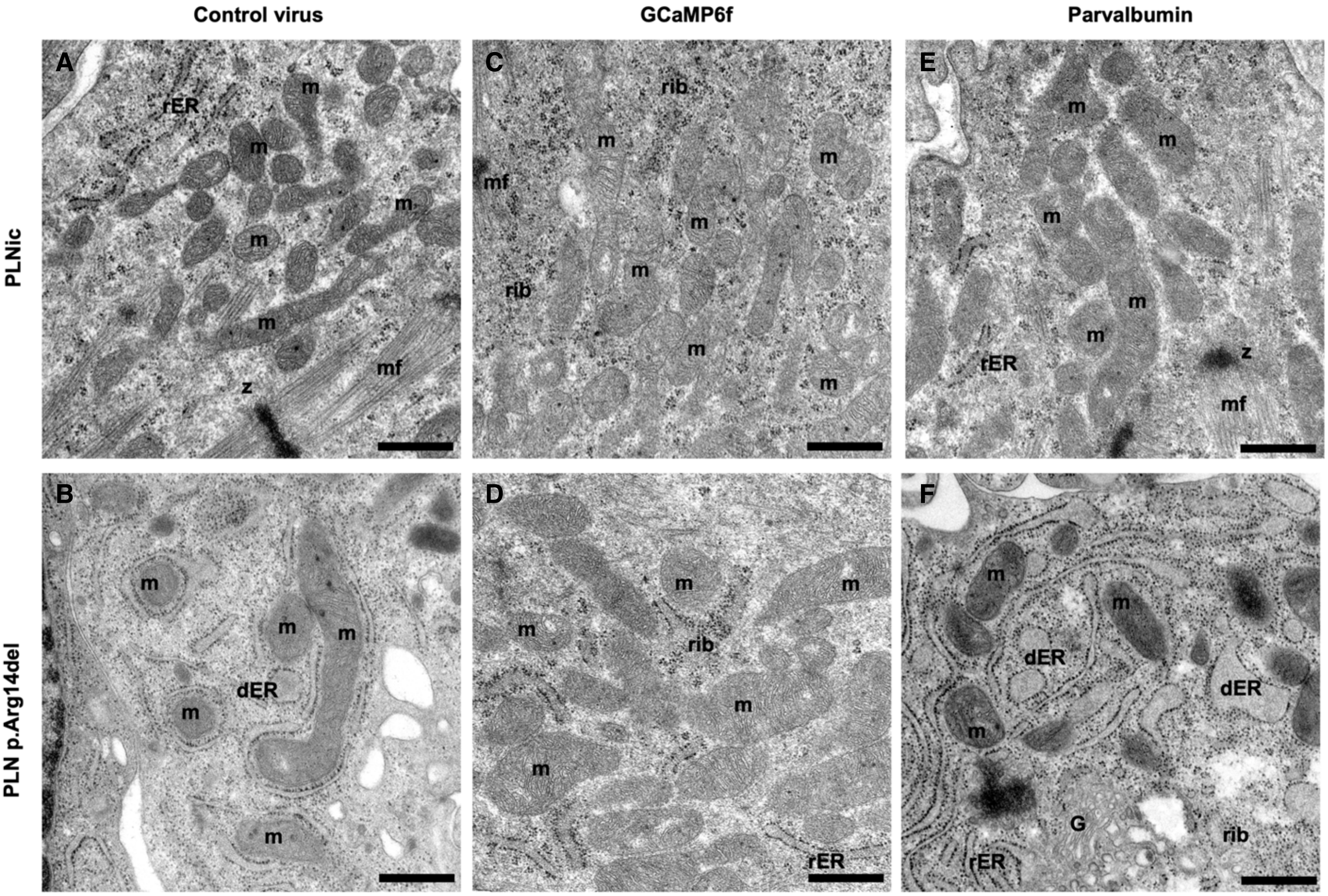

Figure EV3. Transmission electron microscopy.

A–F Transmission electron microscopy of PLNic and PLN p.Arg14del EHTs after transduction with control, GCaMP6f, or parvalbumin virus; mf: myofilaments, z: Z-line, rib: ribosome, m: mitochondria, rER: rough endoplasmic reticulum, dER: dilated endoplasmic reticulum, and G: golgi. Scale bar 500 nm.

 © 2021 The Authors
