# Supplementary material for: Impairment of the ER/mitochondria compartment in human cardiomyocytes with PLN p.Arg14del mutation
Source: EMBO Mol Med. 2021 May 16;13(6):e13074. doi: 10.15252/emmm.202013074 (PMC8185541; doi:10.15252/emmm.202013074)
Supplement: Supplementary file 6 — Movie EV1 [file EMMM-13-e13074-s005.zip › Movie EV1/Legend, Movie EV1.docx]

Movie EV1.-Spontaneous-beating-PLNic-EHT
