# Supplementary material for: Impairment of the ER/mitochondria compartment in human cardiomyocytes with PLN p.Arg14del mutation
Source: EMBO Mol Med. 2021 May 16;13(6):e13074. doi: 10.15252/emmm.202013074 (PMC8185541; doi:10.15252/emmm.202013074)
Supplement: Supplementary file 7 — Movie EV2 [file EMMM-13-e13074-s006.zip › Movie EV2/Legend, Movie EV2.docx]

Movie EV2.-Spontaneous-beating-PLNp.Arg14del-EHT
