# Supplementary figures and images for: Impairment of the ER/mitochondria compartment in human cardiomyocytes with PLN p.Arg14del mutation
Source: EMBO Mol Med. 2021 May 16;13(6):e13074. doi: 10.15252/emmm.202013074 (PMC8185541; doi:10.15252/emmm.202013074)

Figure EV1A

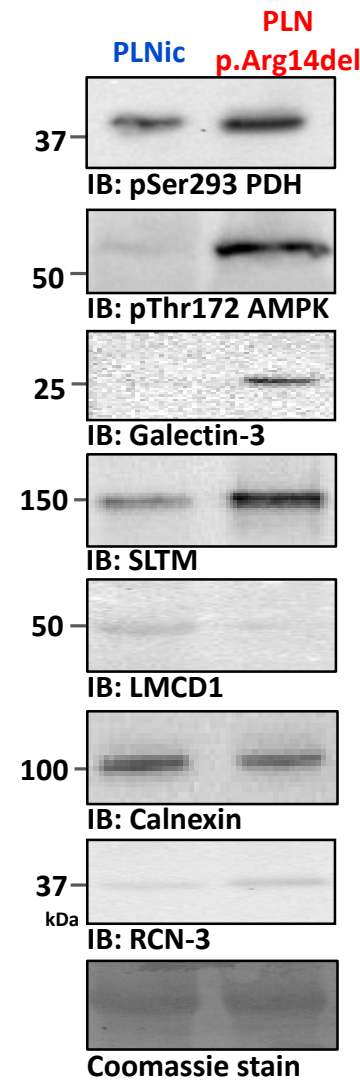

Uncropped Figure EV1A

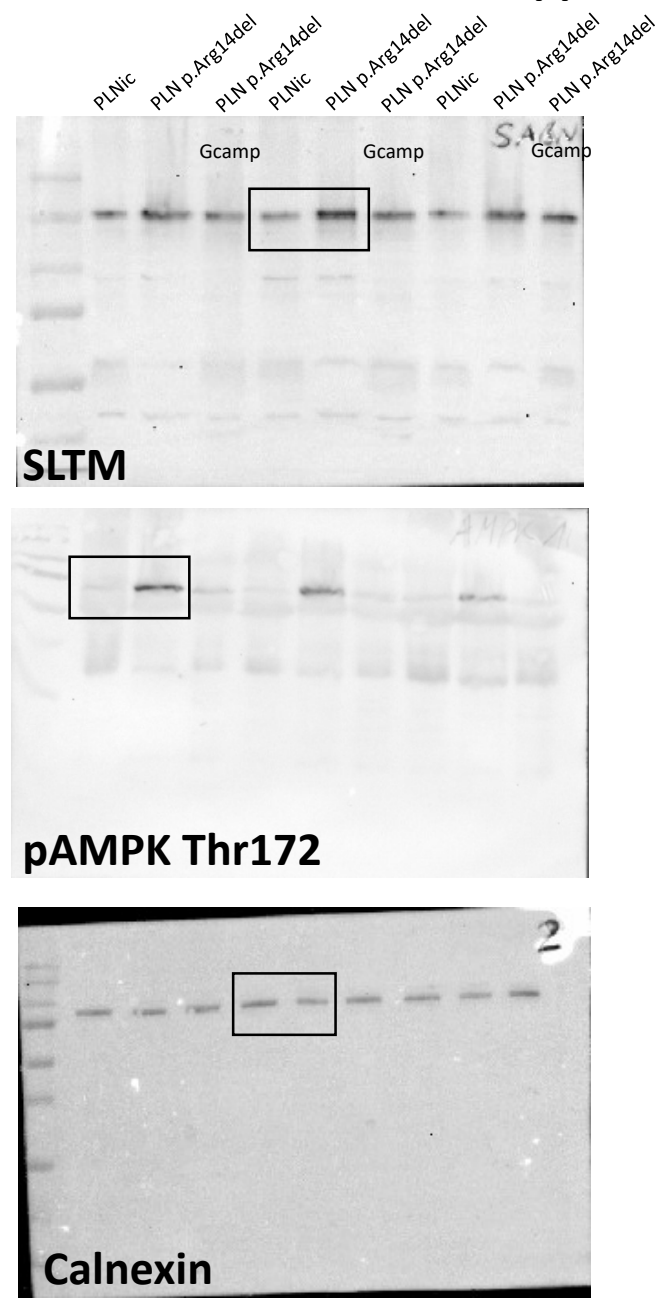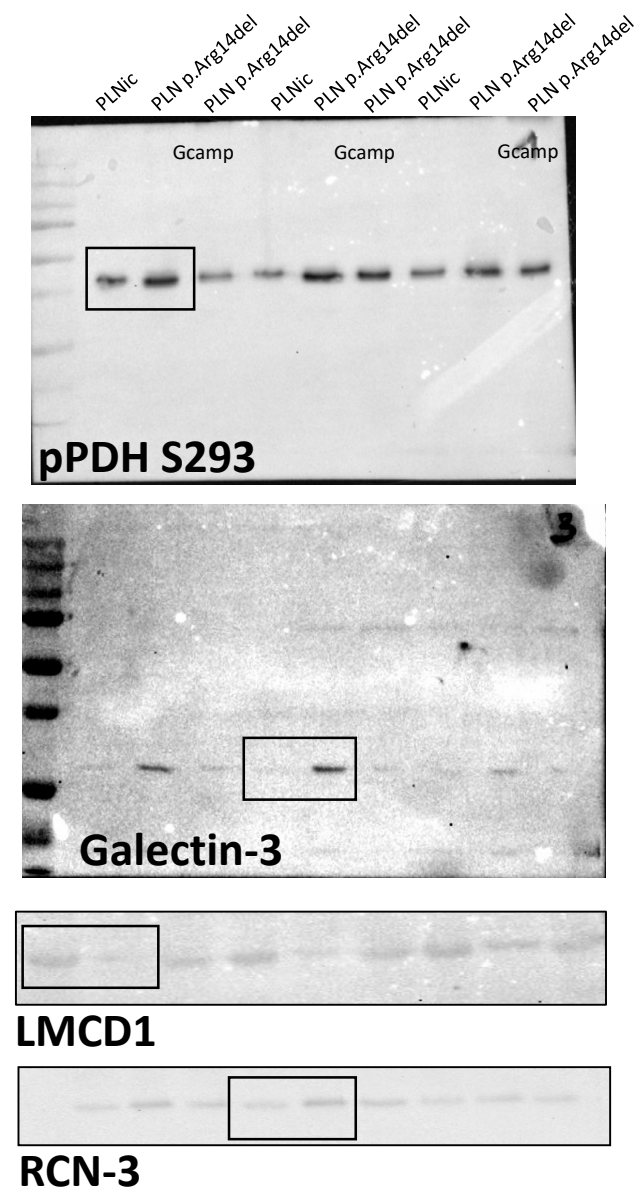

Supplement: Supplementary file 8 — Source Data for Expanded View and Appendix [file EMMM-13-e13074-s010.zip › Source_data_Figure_EV1A_.pdf]

Manuscript Figure 6G

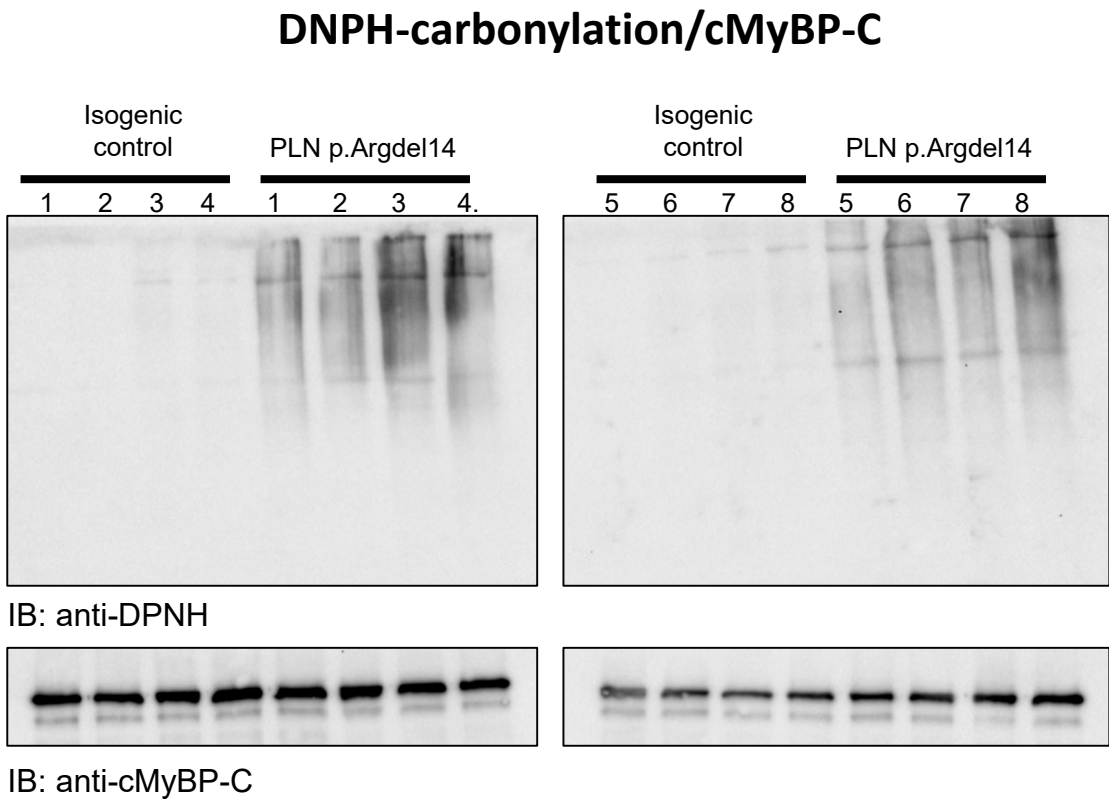

Uncropped Figure 6G

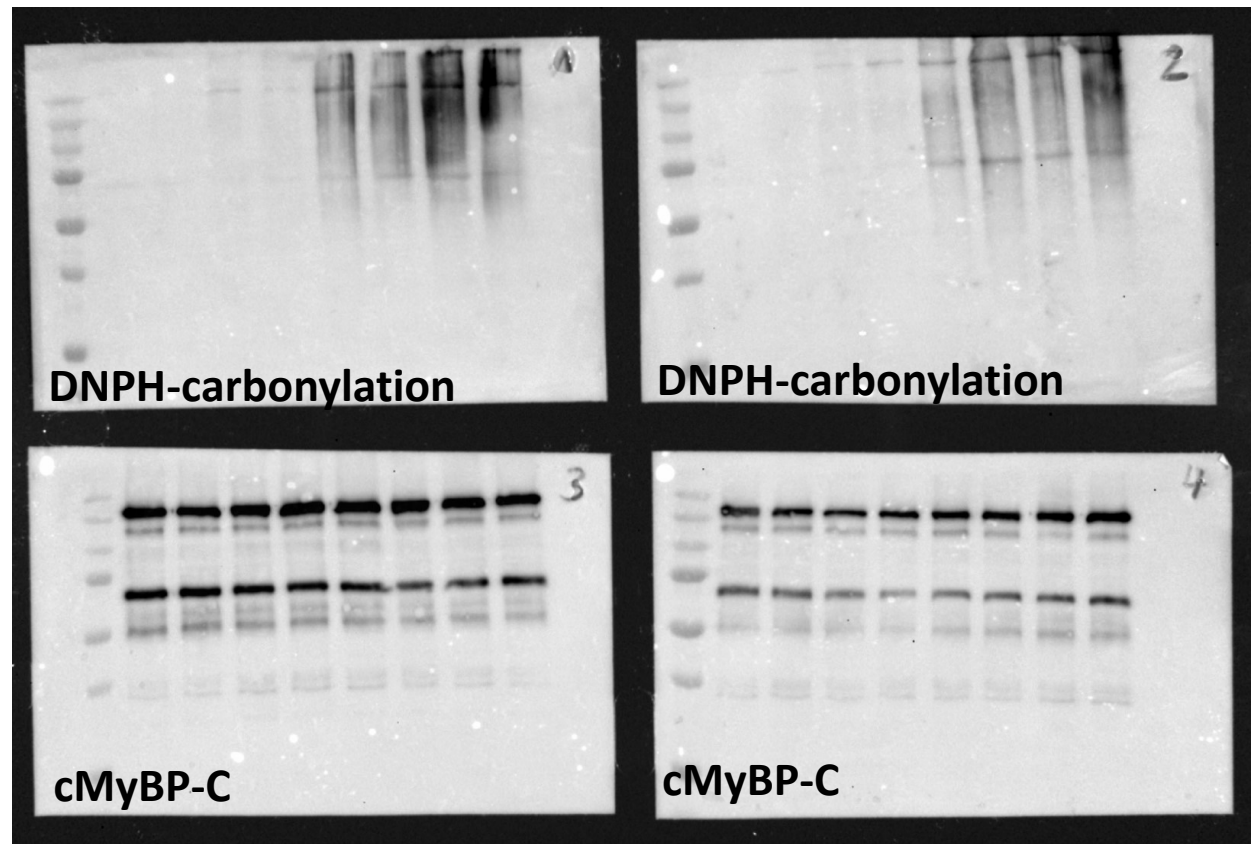

Supplement: Supplementary file 11 — Source Data for Figure 6G [file EMMM-13-e13074-s002.pdf]

Manuscript Figure 8D

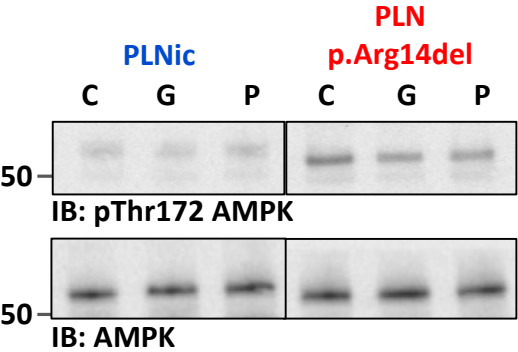

Uncropped Figure 8D

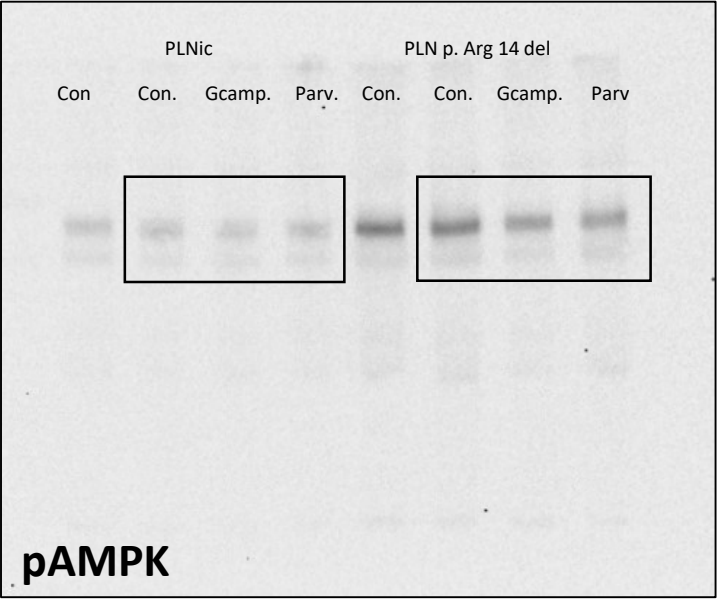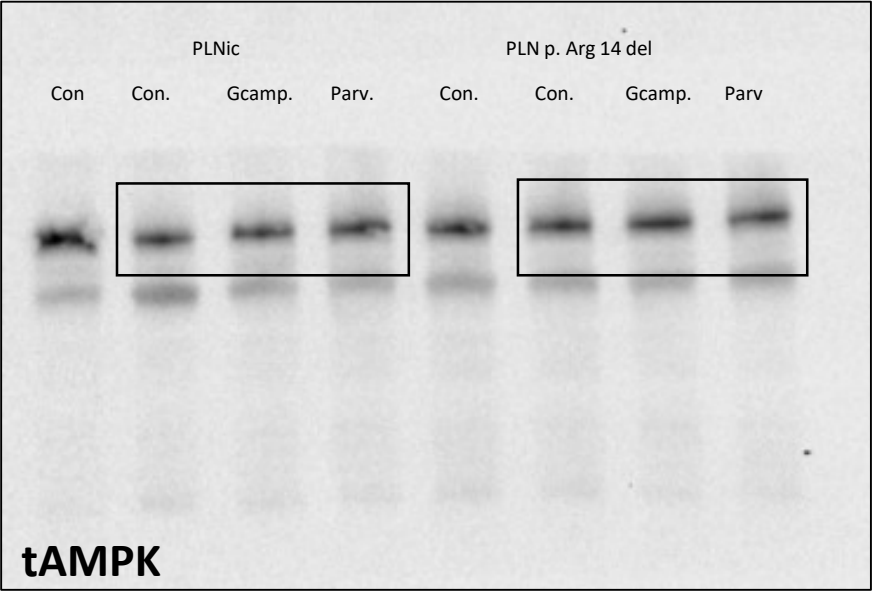

Supplement: Supplementary file 12 — Source Data for Figure 8 [file EMMM-13-e13074-s001.zip › Source_data_Figure_8D.pdf]

### Uncropped Figure 8B

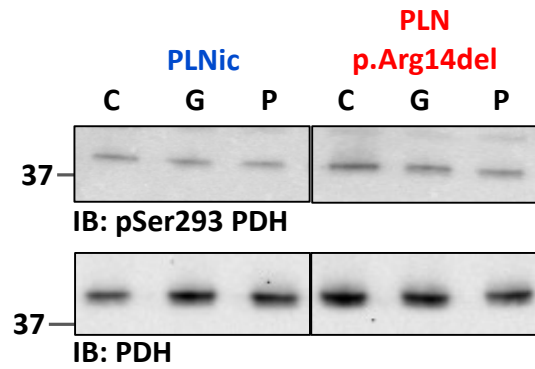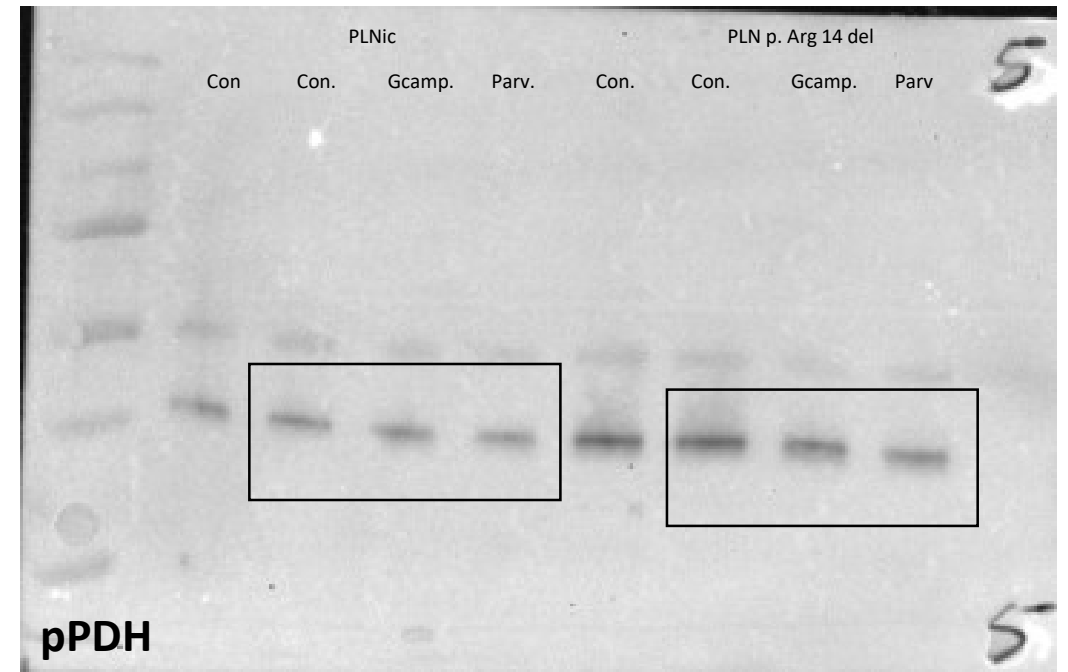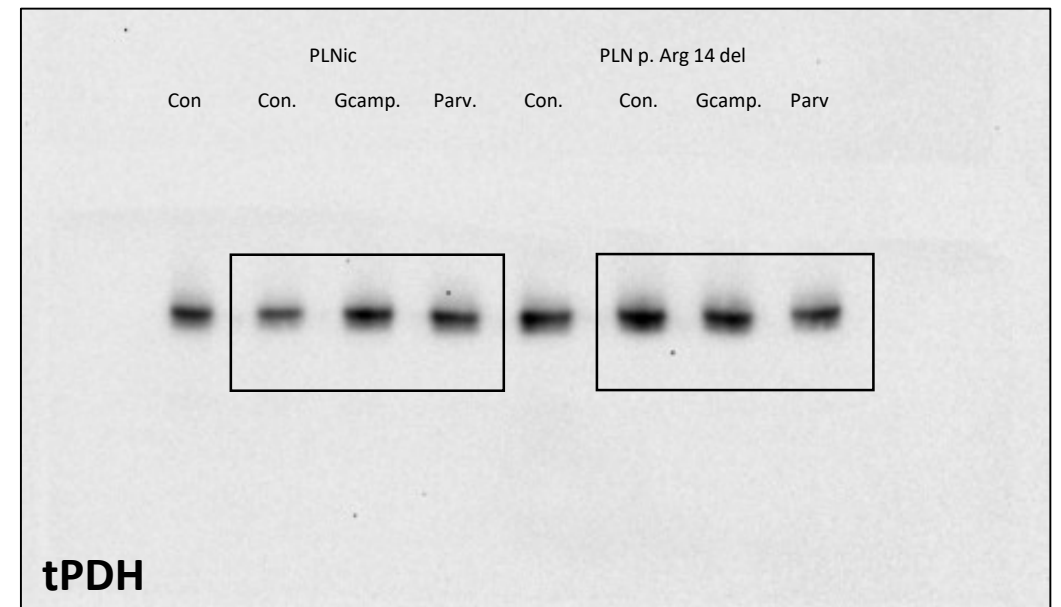

Supplement: Supplementary file 12 — Source Data for Figure 8 [file EMMM-13-e13074-s001.zip › Source_data_Figure_8B.pdf]
