# Supplementary material for: Impairment of the ER/mitochondria compartment in human cardiomyocytes with PLN p.Arg14del mutation
Source: EMBO Mol Med. 2021 May 16;13(6):e13074. doi: 10.15252/emmm.202013074 (PMC8185541; doi:10.15252/emmm.202013074)

Uncropped Western immunoblots

Manuscript Figure 2A

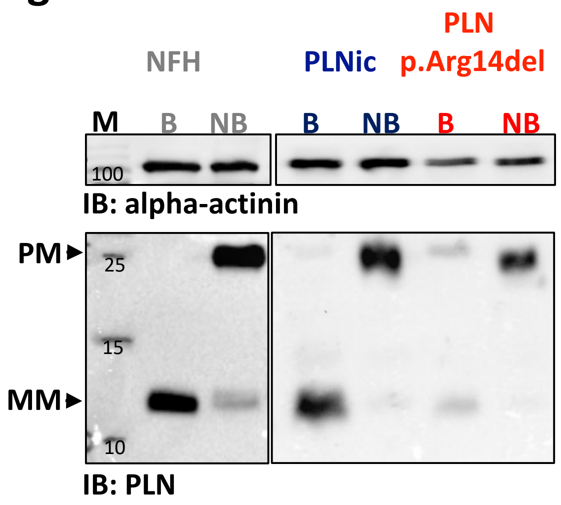

Manuscript Figure 2C

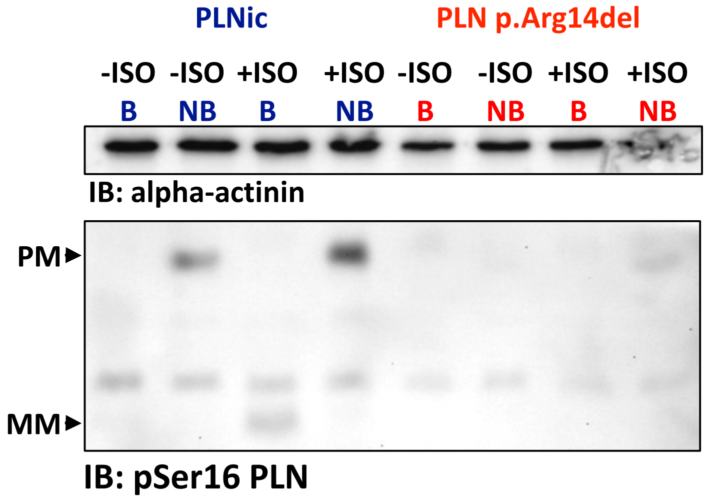

Uncropped Figure 2A

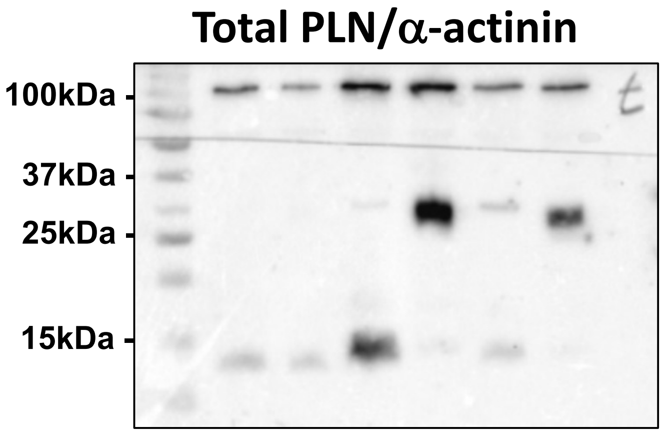

Uncropped Figure 2C

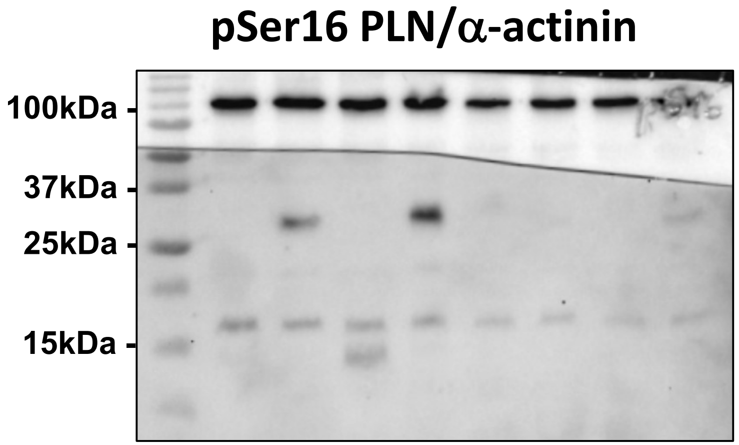

Supplement: Supplementary file 10 — Source Data for Figure 2A,C [file EMMM-13-e13074-s004.pdf]
