# Supplementary material for: Impairment of the ER/mitochondria compartment in human cardiomyocytes with PLN p.Arg14del mutation
Source: EMBO Mol Med. 2021 May 16;13(6):e13074. doi: 10.15252/emmm.202013074 (PMC8185541; doi:10.15252/emmm.202013074)

Appendix Figure S8D

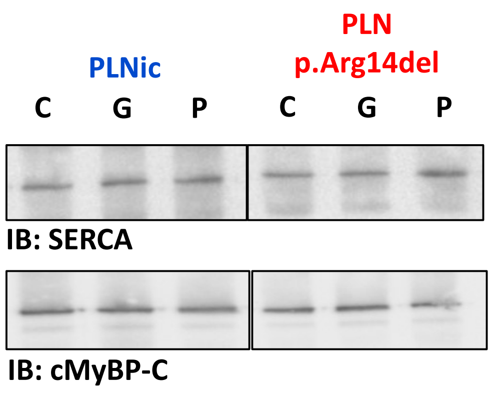

Uncropped Appendix Figure S8D

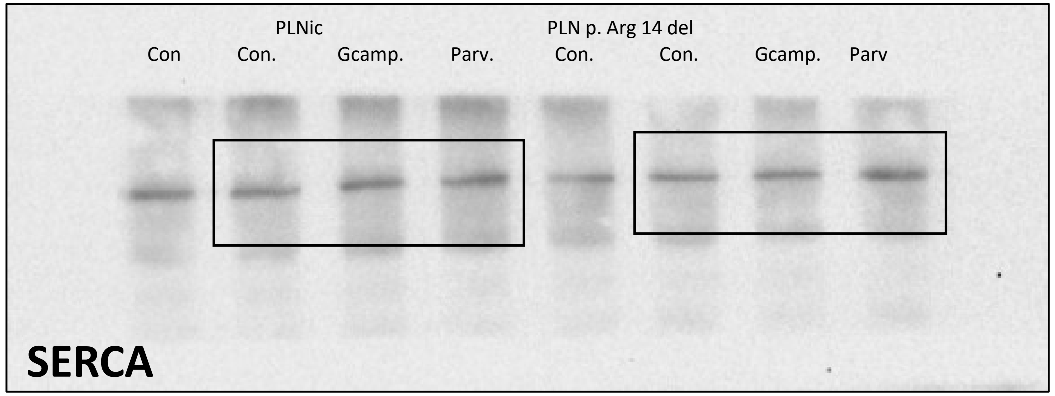

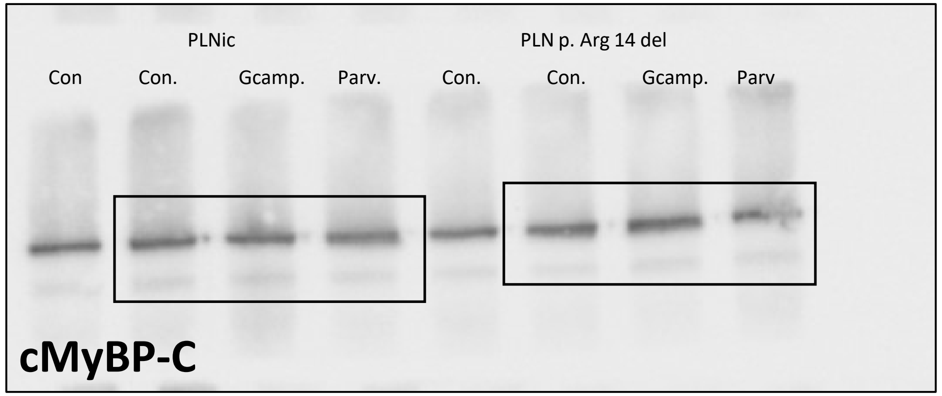

Supplement: Supplementary file 8 — Source Data for Expanded View and Appendix [file EMMM-13-e13074-s010.zip › Source Data Appendix Fig S8.zip!/Source_data_Appendix_Figure_S8D.pdf]
